# Dietary 7-ketocholesterol exacerbates myocardial ischemia–reperfusion injury in mice through monocyte/macrophage-mediated inflammation

**DOI:** 10.1038/s41598-022-19065-z

**Published:** 2022-09-01

**Authors:** Tomoki Uchikawa, Tetsuya Matoba, Takuro Kawahara, Isashi Baba, Shunsuke Katsuki, Jun-ichiro Koga, Yu Hashimoto, Ryo Yamasaki, Ikuyo Ichi, Hidetaka Akita, Hiroyuki Tsutsui

**Affiliations:** 1 Department of Cardiovascular Medicine, Graduate School of Medical Sciences, Kyushu University, 3-1-1, Maidashi, Higashi-Ku, Fukuoka, 812-8582 Japan; 2 Division of Cardiovascular Medicine, Faculty of Medical Sciences, Research Institute of Angiocardiology, Kyushu University, Fukuoka, Japan; 3 Department of Neurology, Graduate School of Medical Sciences, Neurological Institute, Kyushu University, Fukuoka, Japan; 4 Graduate School of Humanities and Science, Ochanomizu University, Tokyo, Japan; 5 Laboratory of DDS Design and Drug Disposition, Graduate School of Pharmaceutical Sciences, Chiba University, Chiba, Japan

**Keywords:** Acute coronary syndromes, Dyslipidaemias

## Abstract

Emerging evidence suggests that 7-ketocholesterol (7-KC), one of the most abundant dietary oxysterols, causes inflammation and cardiovascular diseases. Here we show the deteriorating effects of dietary 7-KC on myocardial ischemia–reperfusion (IR) injury and detailed the molecular mechanisms. A high-fat high-cholesterol diet containing 7-KC (7KWD) for 3 weeks increased the plasma 7-KC level compared with high-fat high-cholesterol diet in mice. In wild-type mice but not in CCR2^−/−^ mice, dietary 7-KC increased the myocardial infarct size after IR. Flow cytometry revealed that the ratio of Ly-6C^high^ inflammatory monocytes to total monocytes was increased in the 7KWD group. Unbiased RNA sequencing using murine primary macrophages revealed that 7-KC regulated the expression of transcripts related to inflammation and cholesterol biosynthesis. We further validated that in vitro, 7-KC induced endoplasmic reticulum stress, mitochondrial reactive oxygen species production, and nuclear factor-kappa B activation, which are associated with increased mRNA levels of proinflammatory cytokines. Administration of N-acetyl-l-cysteine or siRNA-mediated knockdown of PKR-like endoplasmic reticulum kinase or endoplasmic reticulum oxidase 1α suppressed the levels of 7-KC-induced inflammation. Dietary 7-KC exacerbates myocardial IR injury through monocyte/macrophage-mediated inflammation. Endoplasmic reticulum stress and oxidative stress are involved in the 7-KC-induced proinflammatory response in macrophages.

## Introduction

Acute myocardial infarction (AMI) is the most devastating complication of coronary atherosclerosis^1^. Post-infarct left ventricular remodeling causes heart failure and reduces quality of life^2–4^. While primary percutaneous coronary intervention (PCI) reduces mortality after ST-segment elevation myocardial infarction (STEMI)^3,4^, the recent shortening of door-to-balloon time has not reduced in-hospital mortality^5^. Ischemia–reperfusion (IR) injury induces cardiomyocyte death and limits the beneficial effects of PCI^6,7^. Hence, improving the understanding of the detailed molecular mechanisms of myocardial IR injury and developing novel therapeutics targeting IR injury are promising therapy-related strategies to improve the prognosis of STEMI patients.

Myocardial IR injury is mainly due to mitochondrial dysfunction and myocardial inflammation mediated by monocytes/macrophages promoting cell death^6,8,9^. A proinflammatory monocyte subset, Ly-6C^high^ monocytes, exacerbates inflammation and subsequent myocardial IR injury in mice^8–10^. We previously reported that the deficiency of CCR2, a receptor for monocyte chemotactic protein 1 (MCP-1) that mediates monocyte/macrophage chemotaxis, as well as nanoparticle-mediated delivery of an angiotensin II type 1 receptor blocker^11^, a peroxisome proliferator-activated receptor γ agonist^12^, or a toll-like receptor 4 (TLR4) blocker^13^ to the monocytes in the blood and IR heart ameliorated myocardial IR injury by inhibiting the recruitment of Ly-6C^high^ monocytes to the IR heart.

Current lipid-lowering therapies involving statins, ezetimibe, and proprotein convertase subtilisin/kexin type-9 (PCSK-9) inhibitors have been established as standard therapies for coronary artery disease (CAD)^14,15^. There, however, remains a substantial residual risk of CAD even with the administration of these intensive lipid-lowering therapies^16^. Dietary oxysterols produced by nonenzymatically oxidized sterol rings during storage or cooking are an important residual risk factor for CAD^17^. Several oxysterols are reported to induce oxidative stress, inflammation, and cell death^18–21^. We also demonstrated that oxysterols promoted atherosclerosis in mice^22^ and coronary endothelial dysfunction in patients with CAD^23^. The absolute amount of oxysterols in the blood, including 7-ketocholesterol (7-KC), is very low, at approximately 0.1% of the amount of cholesterol^23–25^; however, 7-KC is one of the most common dietary oxysterols and the main component of oxidized cholesterol in oxidized low-density lipoprotein^26,27^ and correlates with cardiovascular outcomes and total mortality in patients with CAD^28^. 7-KC is produced by nonenzymatic oxidization at the C7 position of cholesterol, which changes the hydrogen to a ketone^29^. We previously demonstrated that 7-KC possesses proinflammatory properties in vascular cells, such as promoting endothelial cell proliferation and tissue factor expression in smooth muscle cells^25^. However, the molecular mechanisms by which 7-KC induces inflammation in macrophages are not fully understood, and it has not been clarified whether dietary 7-KC affects myocardial IR injury.

Therefore, we hypothesized that 7-KC can exacerbate myocardial IR injury by promoting monocyte/macrophage-mediated inflammation. The present study used a mouse model of myocardial IR injury to examine the effects of 7-KC on myocardial infarct size and the underlying mechanisms by which 7-KC exacerbates inflammation in the IR myocardium.

## Results

### Dietary 7-KC exacerbated myocardial IR injury through monocyte/macrophage-mediated inflammation

We first quantified plasma lipid and oxysterol levels after 3 weeks of feeding a normal laboratory diet, control western diet (WD), or WD containing 7-KC (7KWD). There were no significant differences in plasma cholesterol and triglyceride levels between the WD group and 7KWD group. Dietary 7-KC, however, markedly increased oxysterol levels, especially the levels of 7-KC and 7β-hydroxycholesterol, a metabolite of 7-KC catalyzed with 11β-hydroxysteroid dehydrogenase type 1 (11β-HSD1)^30^ (Table 1).

**Table 1:** Plasma lipid parameters.

Lipid profiles			
	ND (N = 6)	WD (N = 6)	7KWD (N = 6)
Total cholesterol (mg/dL)	93 ± 5	160 ± 4***	150 ± 8***
LDL cholesterol (mg/dL)	20 ± 1	37 ± 2***	36 ± 1***
HDL cholesterol (mg/dL)	54 ± 2	41 ± 3**	44 ± 2*
Triglyceride (mg/dL)	84 ± 8	23 ± 1***	27 ± 4***

Oxysterols			
	ND (N = 8)	WD (N = 8)	7KWD (N = 8)
7α-hydroxycholesterol	8.9 ± 3.7	41.1 ± 15.1	17.3 ± 6.4
7β-hydroxycholesterol	54.0 ± 20.1	149.8 ± 29.9	1168.0 ± 241.6***^††^
7-ketocholesterol	64.5 ± 12.5	144.7 ± 31.7	399.4 ± 61.9***^††^
5β,6β-epoxycholesterol	14.3 ± 8.5	43.5 ± 11.6	31.2 ± 6.4
4β-hydroxycholesterol	79.0 ± 9.6	385.6 ± 69.2**	457.7 ± 77.4***
cholestan-3β,5α,6β-triol	0.1 ± 0.0	0.3 ± 0.1	0.3 ± 0.2
24-hydroxycholesterol	3.9 ± 1.3	11.6 ± 3.3	25.6 ± 4.4***^†^
25-hydroxycholesterol	18.3 ± 5.2	13.6 ± 3.0	17.5 ± 2.9
27-hydroxycholesterol	22.3 ± 6.2	53.1 ± 11.0*	56.3 ± 6.1*
Total oxysterols	480 ± 61	1067 ± 113	2391 ± 299***^††^

Cholesterol and triglyceride levels are shown in mg/dL. The level of each oxysterol component is shown in ng/mL. Data are expressed as the mean ± SEM and were compared using one-way ANOVA with the post hoc Tukey HSD test. *p < 0.05, **p < 0.01, and ***p < 0.001 versus the ND group. ^†^p < 0.05, and ^††^p < 0.001 versus the WD group. ND, normal laboratory diet; WD, Western diet; 7KWD, WD containing 7-KC.

**Figure 1:**
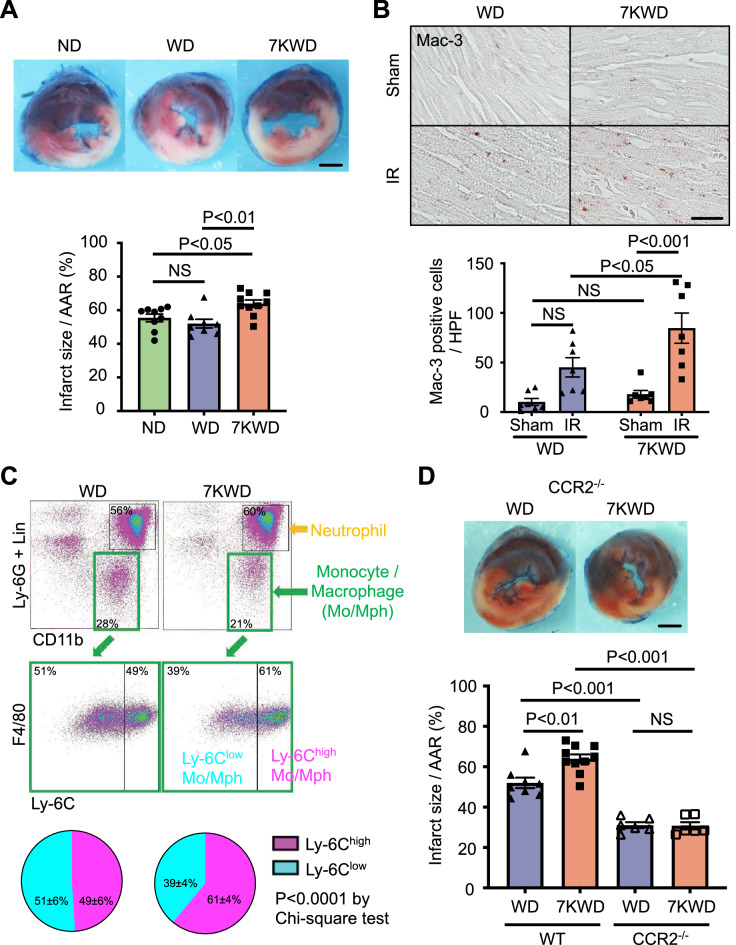
Dietary 7-KC increased the infarct size after myocardial IR through monocyte/macrophage-mediated inflammation. (**A**) Infarct size at 24 h after IR. Scale bar, 1 mm. N = 8–10. The data were analyzed by one-way ANOVA, followed by Tukey’s post-hoc multiple comparison test. (**B**) Macrophages (Mac-3-positive cells) in the heart at 24 h after IR. Scale bar, 20 µm. N = 7. The data were analyzed by one-way ANOVA, followed by Tukey’s post-hoc multiple comparison test. (**C**) Percentages of Ly-6C-high or Ly-6C-low monocytes/macrophages in the heart at 24 h after IR assessed by flow cytometry. N = 6. The data were analyzed by chi-square test. (**D**) Infarct size in WT and CCR2-deficient mice at 24 h after IR. The data of WT group was cited from (**A**) as the control. N = 6. The data were analyzed by one-way ANOVA, followed by Tukey’s post-hoc multiple comparison test. ND, normal laboratory diet; WD, Western diet; 7KWD, WD containing 7-KC; AAR, area at risk; IR, ischemia–reperfusion; Lin, lineage; Mo, monocyte; Mph, macrophage; CCR2^−/−^, CCR2-deficient mice; WT, wild-type mice.

We next examined the effects of dietary 7-KC on myocardial IR injury. As shown in Fig. S1 in the Data Supplement, there were no significant differences in the area at risk (AAR)/LV among the three groups. Importantly, dietary 7-KC increased the infarct size after myocardial IR with no significant effects on blood pressure or heart rate (Figs. 1A, S2 in the Data Supplement). Immunostaining for Mac-3 revealed that the 7KWD increased the accumulation of Mac-3-positive cells compared to the WD after myocardial IR (Fig. 1B). Flow cytometry revealed that the 7KWD increased the ratio of Ly-6C^high^ inflammatory monocytes to total monocytes in the heart after myocardial IR (Figs. 1C, S3 in the Data Supplement). Previous studies have shown that deletion of CCR2 decreases infarct size in a murine myocardial IR injury model, suggesting that monocyte/macrophage-mediated inflammation may play a key role in the mechanisms by which 7-KC exacerbates myocardial IR injury^11^. Immunostaining for MCP-1 and CCR2 revealed that the 7KWD increased the expression of MCP-1 and the accumulation of CCR2-positive cells compared to the WD in the IR heart (Fig. S4 in the Data Supplement). We therefore employed CCR2-deficient mice to clarify the relative importance of monocyte/macrophage-mediated inflammation in the underlying mechanisms. Even though dietary 7-KC increased the infarct size after myocardial IR in wild-type mice (Fig. 1A), CCR2 deficiency abrogated the effect of 7-KC on the infarct size after myocardial IR (Fig. 1D). This result confirmed that dietary 7-KC exacerbated myocardial IR injury primarily through CCR2-positive monocyte/macrophage-mediated inflammation, not through direct effects on cardiomyocytes, after myocardial IR.

### 7-KC facilitated macrophage differentiation into a proinflammatory phenotype and accelerated the macrophage migratory capacity by increasing CCR2 expression

**Figure 2:**
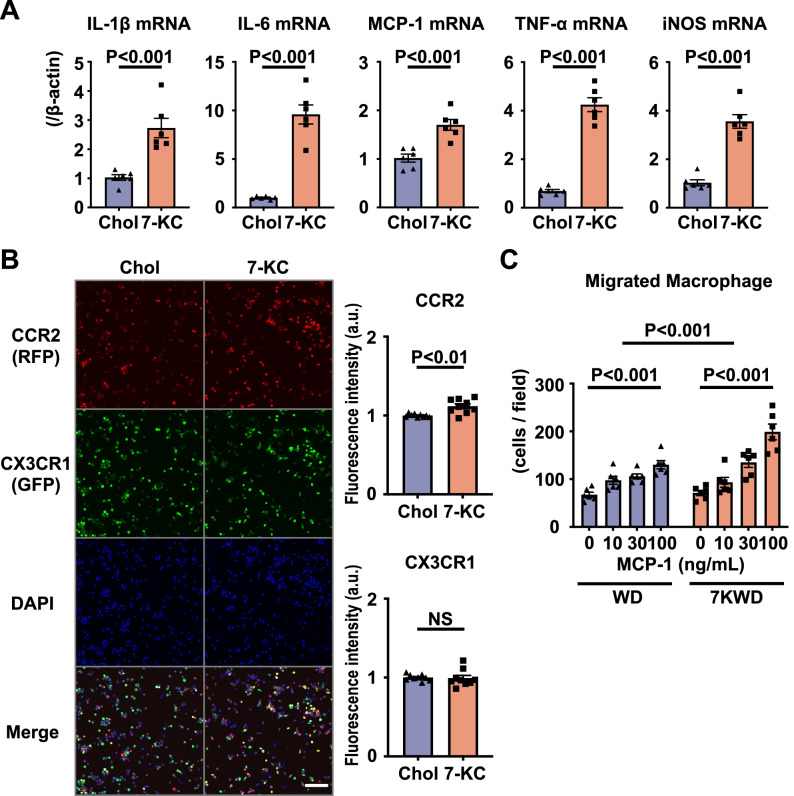
7-KC induced a phenotypic shift toward inflammatory macrophages and increased the chemotactic function of macrophages. (**A**) and (**B**) Bone marrow-derived cells were pretreated with 10 μM cholesterol or 7-KC for 24 h and then stimulated with 10 ng/mL LPS and 10 ng/mL IFN-γ for 24 h. (**A**) mRNA expression of cytokines and iNOS in WT mice. N = 6. The data were analyzed by unpaired t test. (**B**) CCR2 and CX3CR1 expression in CX3CR1-GFP/CCR2-RFP mice. Scale bar, 100 μm. N = 9. The data were analyzed by unpaired t test. (**C**) Chemotactic activity of peritoneal macrophages harvested from WT mice fed a WD or 7KWD for 3 weeks in response to MCP-1. N = 6. The data were analyzed by two-way ANOVA, followed by Tukey’s post-hoc multiple comparison test. Chol, cholesterol; 7-KC, 7-ketocholesterol; WD, Western diet; 7KWD, WD containing 7-KC; LPS, lipopolysaccharide; IFN-γ, interferon-γ.

To elucidate the detailed mechanisms by which 7-KC promotes macrophage-mediated inflammation in the IR heart, we examined the effects of 7-KC on monocyte differentiation. Bone marrow cells isolated from WT mice were cultured with 20 ng/mL macrophage colony-stimulating factor (M-CSF) for 5 days to induce differentiation into bone marrow-derived macrophages (BMDMs). Pretreatment with 7-KC enhanced the induction of proinflammatory factors, including MCP-1 and inducible nitric oxide synthase (iNOS), by 10 ng/mL lipopolysaccharide (LPS) and 10 ng/mL interferon-γ (IFN-γ) compared to control pretreatment with cholesterol (Fig. 2A), suggesting that 7-KC facilitated macrophage differentiation into a proinflammatory phenotype. Next, we used bone marrow cells isolated from CX3CR1-GFP/CCR2-RFP mice to clarify the effects of 7-KC on chemokine receptor expression. In CX3CR1-GFP/CCR2-RFP mice, RFP is coexpressed with CCR2, and GFP is coexpressed with CX3CR1^31^. As shown in Fig. 2B, 7-KC increased CCR2 expression in BMDMs, as determined from the fluorescence intensity of RFP, whereas there were no significant effects on GFP intensity, representing CX3CR1 expression. In vivo flowcytometry analysis, 7KWD increased the ratio of Ly-6C^high^ monocytes in the peripheral blood after myocardial IR compared to WD (data not shown), it was thought 7-KC affected peripheral monocytes and induce polarity shift forward proinflammatory phenotype. Finally, we examined the migratory capacity of peritoneal macrophages isolated from WT mice fed the WD or 7KWD. 7-KC increased the number of migrated macrophages in the 7KWD group compared to that in the WD group (Fig. 2C). Collectively, these results demonstrated that 7-KC promoted macrophage accumulation in the IR heart by increasing the expression of both MCP-1 and CCR2 in macrophages.

### Unbiased transcriptomic analysis revealed 7KC-induced activation of several key proinflammatory signaling pathways

**Figure 3:**
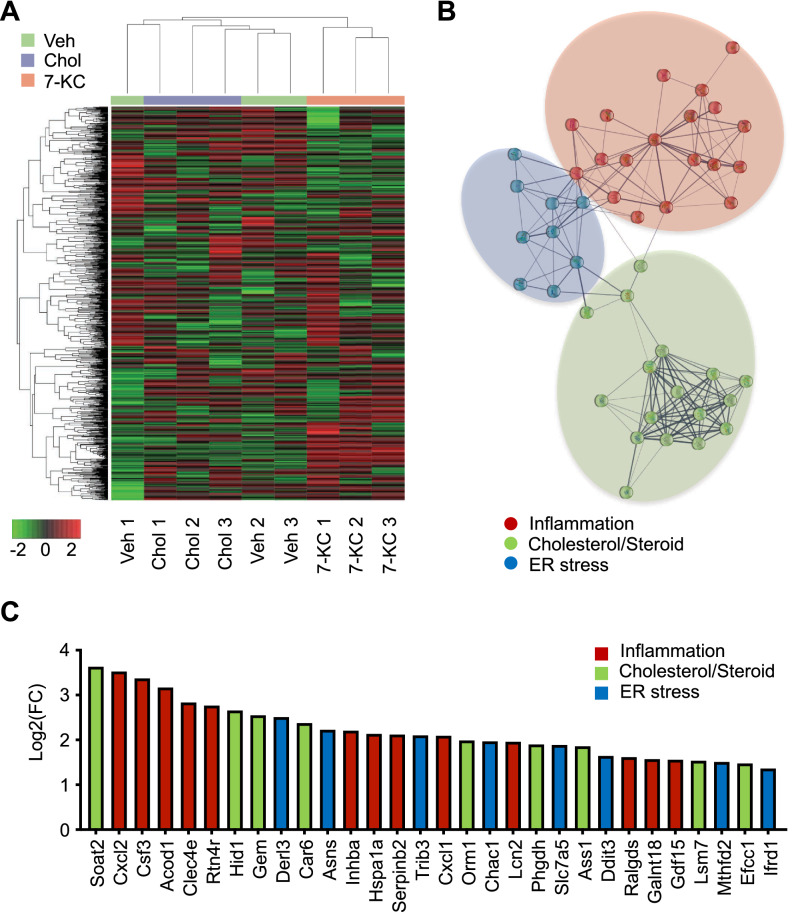
RNA-Seq revealed that 7-KC regulated the expression of transcripts related to inflammation, cholesterol biosynthesis and ER stress. RNA-Seq was performed using RNAs extracted from peritoneal macrophages treated with vehicle, 10 µM cholesterol or 7-KC for 24 h. N = 3. (**A**) Transcriptomic heatmap for the 3 groups. The heatmap was visualized using iDEP software (version 0.94) (http://bioinformatics.sdstate.edu/idep/). (**B**) and (**C**) Network analysis (**B**) and top 30 differentially overexpressed mRNAs (**C**) in macrophages treated with 7-KC compared to those treated with cholesterol. Transcripts were categorized as inflammation- (red), cholesterol/steroid metabolism- (green), and ER stress-associated genes (blue). The network analysis was visualized using STRING software (version 11.5) (https://string-db.org). RNA-Seq, RNA sequencing; Veh, vehicle; Chol, cholesterol; 7-KC, 7-ketocholesterol; ER, endoplasmic reticulum; FC, fold change.

Because in vivo study suggested that monocyte/macrophage-mediated inflammation is responsible for the 7-KC-induced exacerbation of myocardial IR injury, we performed in vitro assays using murine primary macrophages. We explored the effects of 7-KC in an unbiased manner using RNA sequencing (RNA-Seq). RNA-Seq analysis of murine peritoneal macrophages treated with 7-KC revealed differentially expressed transcripts between the 7-KC group and other groups (Fig. 3A). We then performed network analysis using the STRING database (confidence interaction score ≥ 0.400) to extract biological meanings from the differentially expressed transcripts. The network analysis showed a highly clustered network (average local clustering coefficient: 0.555) containing 59 nodes with 169 edges (expected number of edges: 21), suggesting significantly more interaction than expected for a random set of genes of similar size (p value < 1.0 × 10^–16^). The transcriptome was dominated by inflammation, cholesterol/steroid metabolism, and endoplasmic reticulum (ER) stress, as determined by k-means clustering (number of clusters = 3, Fig. 3B). KEGG pathway analysis further confirmed that 7-KC regulated pathways related to inflammation (IL-17 signaling pathway, TNF signaling pathway, cytokine–cytokine receptor interaction, and NF-kappa B signaling pathway), metabolism (steroid biosynthesis and metabolic pathways), and ER stress (protein processing in endoplasmic reticulum), which included the genes encoding Inhibitor of kappa B-alpha (IκBα, *Nfkbia*), tumor necrosis actor-α (TNF-α, *Tnf*), binding immunoglobulin protein (BiP, *Hspa5*) and C/EBP homologous protein (CHOP, *Ddit3*) (Fig. 3C, Table S1 in the Data Supplement).

### ROS mediates 7-KC-induced proinflammatory responses

**Figure 4:**
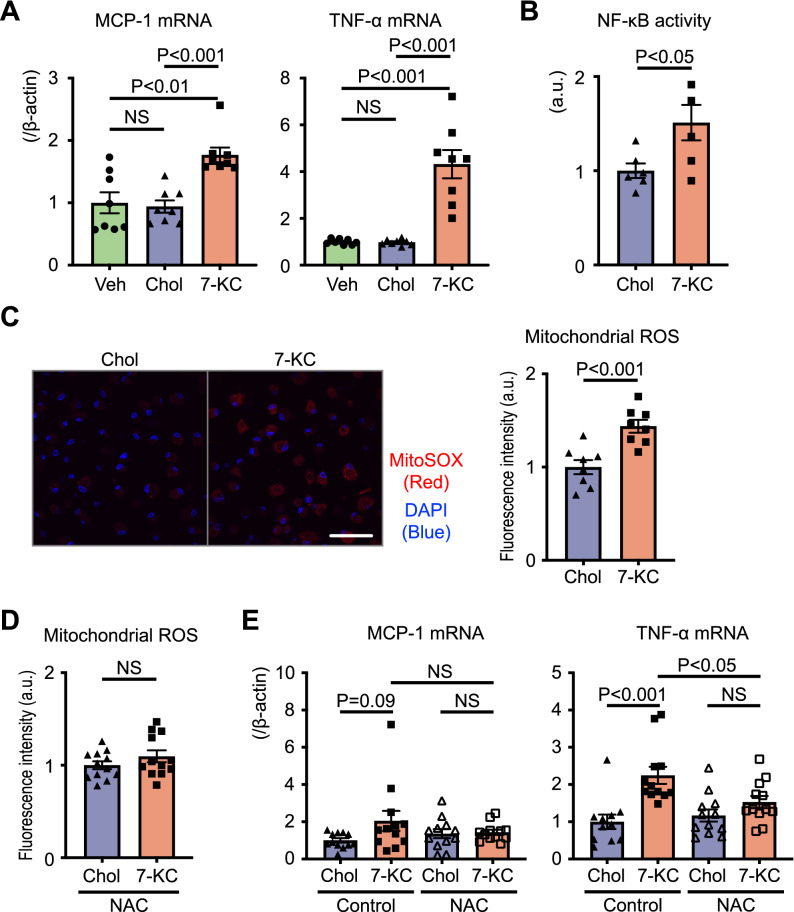
7-KC induced inflammation and ROS production. (**A**–**C**) Peritoneal macrophages were treated with vehicle, 10 µM cholesterol or 7-KC for 24 h. (**A**) Expression of cytokines quantified by RT–PCR. N = 8. The data were analyzed by one-way ANOVA, followed by Tukey’s post-hoc multiple comparison test. (**B**) p65 in the nuclear fraction quantified by ELISA to represent NF-κB activity. N = 6. The data were analyzed by unpaired t test. (**C**) Mitochondrial ROS measured using MitoSOX. N = 8. Scale bar, 50 µm. The data were analyzed by unpaired t test. (**D**) and (**E**) Peritoneal macrophages were pretreated with 5 mM NAC for 1 h before 10 µM cholesterol or 7-KC treatment for 24 h. (**D**) Mitochondrial ROS measured using MitoSOX. N = 12. The data were analyzed by unpaired t test. (**E**) Expression of cytokines quantified by RT–PCR. N = 11–12. The data were analyzed by one-way ANOVA, followed by Tukey’s post-hoc multiple comparison test. Veh, vehicle; Chol, cholesterol; 7-KC, 7-ketocholesterol; ROS, reactive oxygen species; NAC, N-acetyl-l-cysteine.

In cultured macrophages, we confirmed that 7-KC significantly increased the expression of MCP-1 and TNF-α (Fig. 4A). Moreover, 7-KC induced nuclear factor-kappa B (NF-κB) activation, as determined by monitoring p65 nuclear translocation (Fig. 4B). Previous reports have identified 7-KC-induced reactive oxygen species (ROS) production in rat PC-12 cells and mouse J774A.1 cells^32,33^. Furthermore, RNA-Seq revealed that 7-KC regulated the expression of transcripts related to ER stress (Fig. 3B,C), which is regarded as the major inducer of mitochondrial ROS^34^. Therefore, we hypothesized that mitochondrial ROS might play a key role in the mechanisms of 7-KC-induced proinflammatory responses. 7-KC increased ROS in the mitochondrial fraction compared to vehicle or cholesterol (Fig. 4C). N-acetyl-l-cysteine (NAC), a precursor of glutathione that acts as a ROS scavenger, abrogated 7-KC-induced ROS production (Fig. 4D) and TNF-α expression (Fig. 4E). NAC tended to blunt the induction of MCP-1 (Fig. 4E). We also assessed the effect of Mito-TEMPO, mitochondrial-specific ROS scavenger, in cultured macrophages; however, it did not suppress 7-KC-induced inflammatory gene expression (Fig. S5 in the Data Supplement).

Although previous studies have reported that 7-KC increases proinflammatory cytokine levels through TLR4^35^, 7-KC-induced cytokine expression were not decreased in peritoneal macrophages isolated from TLR4-deficient mice (Fig. S6A in the Data Supplement). We also prepared WT peritoneal macrophages with small interfering RNA (siRNA)-mediated knockdown of TLR4. While TLR4-specific siRNA abrogated LPS-induced MCP-1 or TNF-α expression (Fig. S6B in the Data Supplement), 7-KC induced MCP-1 and TNF expression (Fig. S6C in the Data Supplement), which could exclude a major role of TLR4 in 7-KC-induced cytokine expression. Liver X receptors (LXRs) α and β are recognized receptors for oxysterols, including 20(S)-, 22-, 24-, 25-, and 27-hydroxycholesterol and 24, 25-epoxycholesterol^36,37^, while few reports have shown that 7-KC is a weak ligand of LXRs^29,38^. We confirmed that 7-KC increased the expression of LXR target genes related to lipid metabolism, such as ATP binding cassette subfamily A member 1 (ABCA1), ATP binding cassette subfamily G member 1 (ABCG1), and sterol regulatory element-binding protein 1c (SREBP-1c) (Fig. S7 in the Data Supplement). Silencing LXRα and β with siRNA, however, did not inhibit 7-KC-induced proinflammatory responses (Fig. S8A,B in the Data Supplement). These results demonstrated that 7-KC induced monocyte/macrophage-mediated inflammation through mitochondrial ROS-mediated mechanisms but not through LXR or TLR4.

### 7-KC induced ER stress and mitochondrial ROS

**Figure 5:**
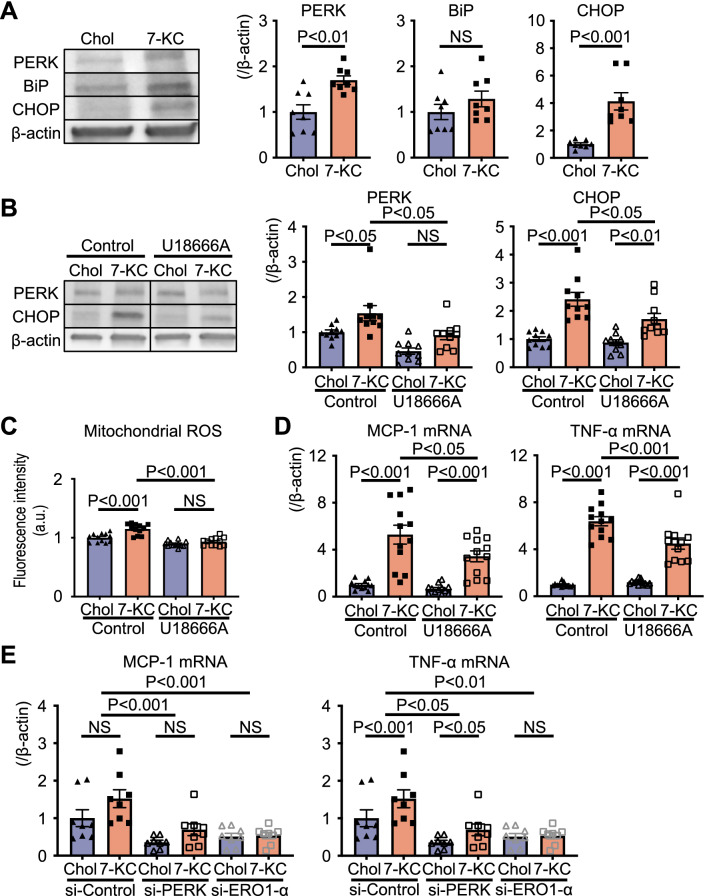
7-KC induced ER stress and subsequently ROS. (**A**) Proteins related to ER stress in peritoneal macrophages treated with 10 µM cholesterol or 7-KC for 24 h. N = 8. The data were analyzed by unpaired t test. Original blots/gels are presented in Supplementary Fig. S7. (**B**–**D**) Effect of the cholesterol transport inhibitor U18666A on peritoneal macrophages pretreated with 3 µM U18666A for 1 h. (**B**) Proteins related to ER stress. N = 10. The data were analyzed by one-way ANOVA, followed by Tukey’s post-hoc multiple comparison test. Original blots/gels are presented in Supplementary Fig. S8. (**C**) Mitochondrial ROS measured using MitoSOX. N = 12. The data were analyzed by one-way ANOVA, followed by Tukey’s post-hoc multiple comparison test. (**D**) Expression of cytokines quantified by RT–PCR. N = 12. The data were analyzed by one-way ANOVA, followed by Tukey’s post-hoc multiple comparison test. (**E**) Effect of siRNA specific for PERK or ERO1-α. Expression of cytokines quantified by RT–PCR. N = 8. The data were analyzed by two-way ANOVA, followed by Tukey’s post-hoc multiple comparison test. Chol, cholesterol; 7-KC, 7-ketocholesterol; PERK, PKR-like endoplasmic reticulum kinase; BiP, binding immunoglobulin protein; CHOP, C/EBP homologous protein; ROS, reactive oxygen species; ER, endoplasmic reticulum; ERO1-α, ER oxidase 1 α; si-Control, nontargeted control siRNA; si-PERK, siRNA against PERK; si-ERO1-α, siRNA against ERO1-α.

ER stress is the major inducer of mitochondrial ROS^34^. Based on our transcriptomic analysis (Fig. 3), ER stress was identified as one of the key signaling pathways in the 7KC-induced response. Western blotting showed that 7-KC significantly increased the levels of PKR-like endoplasmic reticulum kinase (PERK) and CHOP, molecules associated with ER stress (Figs. 5A, S9 in the Data Supplement). Previous studies have demonstrated that U18666A, a blocker of free cholesterol transport from the plasma membrane to the ER, decreases cholesterol-induced ER stress^39^. Thus, we examined the effect of U18666A on 7-KC-induced ER stress. The 7-KC-induced expression of molecules related to ER stress, production of mitochondrial ROS and expression of proinflammatory cytokines were significantly decreased by U18666A (Figs. 5B–D, S10 in the Data Supplement). ER oxidase 1 α (ERO1-α), an important source of ROS during ER stress^40,41^, was found to mediate 7-KC-induced proinflammatory cytokine expression in peritoneal macrophages transfected with PERK- or ERO1-α-specific siRNA (Figs. 5E, S11 in the Data Supplement).

## Discussion

The present study investigated the effects of exogenous 7-KC in a mouse myocardial IR injury model and in cultured monocytes/macrophages. The novel findings included the following: (1) we created a murine model fed with 7-KC and underwent myocardial IR, (2) dietary 7-KC exacerbated myocardial IR injury through CCR2-positive monocyte/macrophage-mediated inflammation in mice, (3) 7-KC induced the differentiation and activation of monocytes/macrophages, and (4) ER stress and mitochondrial ROS production were involved in the inflammation observed in macrophages treated with 7-KC.

In our study, we reported the adverse effect of dietary 7-KC in the WT murine IR model for the first time. The plasma 7-KC level in our in vivo murine model fed with 7KWD reached at approximately 400 ng/mL, which was similar to 7-KC levels in CAD patients, approximately 10 to 500 ng/mL, who were not familial hypercholesterolemia in previous clinical studies^23,28^. Therefore, results of our in vivo study are relevant to provide mechanistic insights into clinical observations that high 7-KC was associated with worse CAD outcomes.

In this study, we employed unbiased transcriptomic analysis and determined the molecular mechanisms of 7-KC-induced inflammation in monocytes/macrophages. Huang et al. previously reported that 7-KC induced inflammation in human retinal pigment epithelium through a TLR4-dependent pathway by using a TLR4 inhibitor^35^. However, in our study, 7-KC increased proinflammatory cytokine expression in peritoneal macrophages isolated from TLR4-deficient mice (Fig. S6A). We also evaluated the effect of TLR4 knockdown to 7-KC-induced inflammation in peritoneal macrophages using siRNA transfection, and showed that TLR4 knockdown did not influence 7-KC-induced MCP-1 or TNF-α expression (Fig. S6C), excluding a role for TLR4 in 7-KC-induced inflammation in macrophages. This difference may be because of the difference in cell type or the method of TLR4 inhibition. We revealed that 7-KC induced the expression of transcripts related to metabolic systems by RNA-Seq and confirmed that 7-KC increased the expression of LXR target genes such as ABCA1, ABCG1 and SREBP-1c. Although modulating LXRs might contribute to inflammatory gene expression by interfering with the LXR-mediated anti-inflammatory effects including the transrepression of inflammatory genes^42,43^, the induction of proinflammatory cytokines was LXR-independent and mediated by ER stress and redox signaling. As 7-KC is a weak agonist of LXRs, the proinflammatory effect exceeds LXR-mediated anti-inflammatory effects in macrophages studied. Pedruzzi et al. reported that 7-KC induced NADPH oxidase 4 (NOX4) and ROS production in smooth muscle cells^19^. Although 7-KC induced ROS production, RNA-Seq showed that the expression of NOX4 did not increase with 7-KC treatment in macrophages (data not shown). In this study, we showed that 7-KC increased mitochondrial ROS in macrophages (Fig. 4C), and that 7-KC-induced inflammatory gene expression was blocked by NAC but not Mito-TEMPO (Figs. 4E, S5). Mito-TEMPO works as a mitochondrial superoxide dismutase (SOD) mimetic that converts superoxide into hydrogen peroxide. SOD is reported to increase hydrogen peroxide and activate redox signaling depending on conditions^44^. NAC is known to induce glutathione peroxidase activity, as well as directly react with hydrogen peroxide, hypochlorous acid, and hydroxyl radicals^45^, which might counteract with downstream redox signaling to induce inflammatory genes.

ER stress is regarded as the major inducer of mitochondrial ROS, and ERO1-α regulated by CHOP plays an important role as a protein disulfide oxidase in ER stress-induced ROS production^40,41^. ERO1-α mediates the transfer of electrons from protein disulfide isomerase to oxygen, resulting in ROS production. In addition, ERO1-α increases inositol-1,4,5-trisphosphate receptor-mediated Ca^2+^ release from the ER, and then the released Ca^2+^ accumulates in the mitochondria and disrupts the electron transport chain, leading to the production of mitochondrial ROS. We employed U18666A, a cholesterol transport inhibitor, and siRNA against PERK or ERO1-α and found that inhibition of ER stress decreased 7-KC-induced ROS production; thus, it seemed that 7-KC increased ROS production mainly through ER stress, not through NOX4 upregulation in macrophages.

In the current era of early reperfusion therapy, myocardial IR injury is a crucial and residual therapeutic target for reducing infarct size and improving long-term prognosis. No medical therapies, however, are currently available for the treatment of myocardial IR injury. We have shown that inflammation in the heart, which is primarily mediated by monocytes/macrophages, synergistically plays a role in IR injury, even in the absence of mitochondrial permeability transition pore opening^11,46^. In the present study, dietary 7-KC increased the expression of MCP-1 and the accumulation of CCR2-positve cells in the IR heart, and CCR2 deficiency abrogated the effect of 7-KC on the infarct size after myocardial IR; therefore, we concluded that 7-KC exacerbated myocardial IR injury primarily through monocyte/macrophage-mediated inflammation. Though myocardial IR injury increased infiltration of neutrophils in the ischemic heart, 7KWD did not increase neutrophils in the heart after myocardial IR compared to WD (data not shown). Our previous study using anti-Ly6G antibody, elimination of neutrophils did not affect myocardial IR injury in the same mouse model^11^, thus we consider that 7-KC exacerbated myocardial IR injury mostly through monocyte/macrophage. We also reported some findings related to inflammation: (1) 7-KC increased the expression of CCR2 in BMDMs, (2) 7-KC increased the chemotactic activity of macrophages, and (3) 7-KC increased the expression of MCP-1 and TNF-α. These findings suggest that 7-KC promotes macrophage accumulation in the IR heart by increasing MCP-1 and CCR2 expression on macrophages and that these macrophages release cytotoxic factors, such as TNF-α, resulting in a larger infarct size. Song et al. reported that a high plasma 7-KC level was related to a high rate of cardiovascular events, including AMI^28^, which might result from 7-KC-induced chronic activation of circulating CCR2-positive monocytes. Based on our findings, 7-KC is a potential therapeutic target in patients with AMI, and targeting 7-KC may ameliorate myocardial inflammation and IR injury. We previously demonstrated that ezetimibe, a cholesterol transporter Niemann-pick C1-like 1 inhibitor, suppresses 7-KC absorption in the intestine and decreases the serum 7-KC concentration^25^. Therefore, our findings could be translated into practice through clinical trials. Furthermore, because it has been reported that 7-KC is related to other cardiometabolic diseases, such as nonalcoholic fatty liver disease^47^ and age-related macular degeneration^48^, 7-KC could also be a therapeutic target in such diseases. It is noteworthy that the 7-KC level was significantly increased in heart tissue, not only in the blood (data not shown), and the related roles should be explored in other heart diseases.

There are some limitations to the present study. Firstly, in vitro assays performed with 7-KC may not represent the activity of 7-KC in an in vivo environment, in which 7-KC is contained in lipoproteins such as low-density lipoprotein cholesterol in the circulating blood. Therefore, the kinetics of 7-KC, including interactions with the cell membrane and receptors, might be different in in vivo situations. Secondly, since we focused on the acute phase of myocardial IR injury in this study, we did not evaluate the role of wound-healing macrophages and their efferocytosis that might contribute to left ventricular remodeling after myocardial infarction. Future study is needed to evaluate the effect of 7-KC on efferocytosis.

In conclusion, we showed for the first time that dietary 7-KC exacerbates myocardial IR injury through CCR2-positive macrophage-mediated inflammation. ER stress and oxidative stress are key processes involved in the 7-KC-induced proinflammatory responses in macrophages.

## Methods

### Experimental animals

Adult male C57BL/6J mice (10 weeks old) (Japan SLC Inc., Shizuoka, Japan), CCR2-deficient mice on the C57BL/6J background (10 weeks old) (CLEA Japan Inc., Tokyo, Japan), TLR4-deficient mice on the C57BL/6J background (10 weeks old) (Oriental Bio Service Inc., Kyoto, Japan) and CX3CR1-GFP/CCR2-RFP mice on the C57BL/6J background (10 weeks old) (gifted by Dr. R. Yamasaki) were used in this study^31^. All mice were maintained on a 12-h light/dark cycle and fed either a normal laboratory diet or a high-fat high-cholesterol diet and water ad libitum. The study protocol was reviewed and approved by the Committee on the Ethics of Animal Experiments, Kyushu University Faculty of Medicine, which is compatible with the ARRIVE guidelines. All the experiments were conducted following the American Physiological Society guidelines and the NIH Guide for the Care and Use of Laboratory Animals, 8th edition.

### Measurement of plasma lipid levels

Plasma was collected for cholesterol, oxysterol, and triglyceride measurements. Total cholesterol, low-density lipoprotein cholesterol, high-density lipoprotein cholesterol, and triglycerides were measured using enzymatic assay kits (FUJIFILM Wako Pure Chemical Co., Osaka, Japan). Lipids extracted plasma were saponified at room temperature overnight in the dark. Unsaponified lipids were applied to a Sep-Pak Vac silica cartridge (Waters Corporation, Milford, MA) to separate oxysterols and sterols^49^. Oxysterols, including 7-KC, were converted into trimethylsilyl ethers in a mixture of trimethylchlorosilane, 1,1,1,3,3,3-hexamethyldisilazane, and dried pyridine (1:3:9, v:v:v) for 30 min at room temperature. Oxysterols were measured by gas chromatography–mass spectrometry using a GCMS-QP2010 system (Shimadzu Co., Kyoto, Japan) equipped with an SPB-1–fused silica capillary column of 60 m × 0.25 mm and 0.25-μm phase thickness (Supelco Inc., Bellefonte, PA).

### Mouse myocardial IR injury model

Mice were fed a normal laboratory diet (CRF-1), WD, or 7KWD for 3 weeks, and then surgery causing myocardial IR was performed. The WD contained 40% fat, 1% cholesterol and 0.5% cholic acid, and the 7KWD contained 40% fat, 0.9% cholesterol, 0.1% 7-ketocholesterol and 0.5% cholic acid. All these diets were purchased from ORIENTAL YEAST CO., Ltd. (Tokyo, Japan), and supplemental 7-KC was purchased from Sigma–Aldrich (St. Louis, MO). A murine model of myocardial IR injury was established as previously described^50,51^. Mice were kept warm on a heating pad and anesthetized with 1.5% isoflurane in 2 L/min oxygen. Subsequently, the mice were intubated and ventilated with a respirator. The heart was exposed by a left thoracotomy and pericardiotomy. The left anterior descending artery (LAD) was temporarily ligated with an 8–0 nylon suture and silicon tube (1 mm OD) for 30 min, and then the silicon tube was removed to restart the blood flow.

### Preparation of primary macrophages

As previously described, murine peritoneal and BMDMs were prepared for in vitro assays^52^. Peritoneal macrophages were collected as described below. Brewer’s thioglycolate medium (BD Diagnostic Systems, Sparks, MD) was injected into the murine peritoneal cavity 3 days before macrophage collection. Ice-cold phosphate-buffered saline (PBS) was injected into the peritoneal cavity, and cells were harvested. Red blood cells were lysed with ammonium-chloride-potassium lysis buffer (Thermo Fisher Scientific Inc., Cleveland, OH). Then, the cells were washed with PBS, resuspended in Roswell Park Memorial Institute 1640 (RPMI-1640) medium (Sigma–Aldrich) supplemented with 10% fetal bovine serum (FBS) (Sigma–Aldrich) and 1% penicillin/streptomycin and plated in culture plates. One hour later, the nonadherent cells were removed, and the remaining adherent cells were cultured further in fresh medium. Bone marrow-derived macrophages were harvested from the tibiae and femurs of donor mice. After red blood cell lysis and washing, the cells were resuspended and cultured in RPMI-1640 medium supplemented with 10% FBS, 1% penicillin/streptomycin, and 20 ng/mL M-CSF (R&D Systems, Minneapolis, MN).

### siRNA transfection

ON-TARGETplus Non-targeting Control Pool or ON-TARGETplus SMARTPool siRNAs targeting mouse *Tlr4*, *Nr1h3*, *Nr1h2*, *Eif2ak3*, or *Ero1l* were purchased from Horizon Discovery Ltd. (Cambridge, UK). All siRNAs were packaged in lipid nanoparticles (LNPs) and transfected into cells. Cultured cells were transfected with siRNA for 24 h and then treated with cholesterol or 7-KC 12 h after transfection was completed. siRNA-loaded LNPs were formulated as previously described^53^. Briefly, 400 μL of lipid solution containing 1.875 mM ssPalmO-Phe (Product# COATSOME^®^ SS-OP, NOF CORPORATION, Tokyo, Japan), 450 μM polyethylene glycol-dimyristoylglycerol (Product# SUNBRIGHT^®^ GM-020, NOF CORPORATION), and 1.875 mM cholesterol (Sigma–Aldrich) in ethanol was prepared. Then, 10 μg of siRNA in 124 μL of 20 mM malic acid buffer (pH 3.0) was gradually added to the lipid solution under vigorous mixing. The mixed solution was diluted with 1 mL of malic acid buffer and then mixed vigorously with 3 mL of PBS. This diluted solution was subjected to ultrafiltration with Amicon Ultra-4 (Merck Millipore, Billerica, MA) two or more times. The encapsulation efficiency and recovery ratio of siRNA in this LNP solution were measured with a RiboGreen assay (Thermo Fisher Scientific Inc.) as previously described^54^.

### Quantification of myocardial infarction

Twenty-four hours after reperfusion, mice were anesthetized with 1.5% isoflurane in 2 L/min oxygen and intubated. After ligating the LAD again, 2% Evans blue dye (Sigma–Aldrich) was injected into the inferior vena cava to dye the nonischemic area of the heart. Hearts were harvested and washed by saline perfusion. The harvested hearts were frozen in liquid nitrogen and cut into sequential cross-sections at a thickness of 1 mm. The sections were incubated with 1.5% 2,3,5-triphenyltetrazolium chloride (TTC) (Sigma–Aldrich) for 20 min at 37 °C. After dyeing with TTC, the sections were placed in 10% formaldehyde overnight. Then, the sections were photographed under a microscope. The infarcted area (TTC negative/Evans blue negative, white), the noninfarcted area within the AAR (TTC positive/Evans blue negative, red), the nonischemic area (TTC positive/Evans blue positive, purple), and the AAR (Evans blue negative, red and white) were quantified using ImageJ software (version 1.51 h). The infarct area/AAR was quantified and compared among each group. In all experiments of myocardial IR injury, we followed a standard protocol to evaluate the differences in myocardial IR injury^13,51^.

### Immunohistochemistry

Paraffin sections of the heart were prepared for immunohistochemistry. Twenty-four hours after reperfusion, hearts were harvested, fixed in 10% formaldehyde for 24 h, and then embedded in paraffin. Serial cross-sections of the heart cut at a thickness of 5 μm at the midventricular level were used for analysis. For mac-3 staining, after blocking with 2.5% goat serum (ImmPRESS, Goat Anti-Rat IgG Polymer Kit, Peroxidase, Vector Laboratories, Burlingame, CA), the sections were subjected to immunostaining for macrophages using an anti-mouse Mac-3 antibody (M3/84, 1:50, BD Pharmingen, San Diego, CA). After an overnight incubation with the primary antibody at 4 °C, the sections were washed 3 times with PBS and incubated with peroxidase-conjugated anti-rat IgG at room temperature for 30 min. Then, the sections were washed 3 times with PBS and incubated with a peroxidase substrate (ImmPACT, AMEC Red Peroxidase Substrate Kit, Vector Laboratories) for 10 min. After counterstaining with hematoxylin, the sections were imaged with a stereomicroscope. Mac-3-positive cells were counted using ImageJ software (version 1.51 h). For MCP-1 staining, we used 5% skim milk (MEGMILK SNOW BRAND CO., Ltd., Tokyo, Japan) for blocking, anti-MCP-1 antibody (R-17, 1:200, Santa Cruz Biotechnology, Inc., Dallas, TX) as primary antibody, Histofine Simple Stain Mouse MAX PO(G) (NICHIREI BIOSCIENCES, Inc., Tokyo, Japan) as secondary antibody, and Dako REAL EnVision-HRP Rabbit-Mouse (Agilent Technologies, Inc., Santa Clara, CA) for detection. For CCR2 staining, we used 5% skim milk for blocking, anti-CCR2 antibody (EPR19698, 1:100, abcam, Cambridge, UK) as primary antibody, Dako EnVision + System HRP Labelled Polymer Anti-Rabbit (Agilent Technologies, Inc.) as secondary antibody, and Dako REAL EnVision-HRP Rabbit-Mouse for detection.

### Flow cytometry

Twenty-four hours after reperfusion, hearts were harvested, cut into small pieces, and digested with a cocktail of 450 U/mL collagenase type I, 125 U/mL collagenase type XI, 60 U/mL DNase I, and 60 U/mL hyaluronidase (Sigma–Aldrich) in PBS containing 20 mM 4-(2-hydroxyethyl)piperazine-1-ethanesulfonic acid (HEPES) at 37 °C for 40 min. The cell suspensions were washed with PBS, and Fc receptors were blocked with anti-CD16/32 antibodies (BD Pharmingen). After blocking, the cell suspensions were incubated with a cocktail of antibodies against leukocytes (anti-CD45-PE-Cy7, 30-F11), myeloid cells (anti-CD11b-APC-Cy7, M1/70), B cells (anti-B220-PE, RA3-6B2), T cells (anti-CD90.2-PE, 53-2.1), NK cells (anti-CD49b-PE, DX5; NK1.1-PE, PK136), granulocytes (anti-Ly-6G-PE, 1A8), monocyte subsets (anti-Ly-6C-FITC, AL-21), and macrophages (anti-F4/80-Alexa Fluor 647, T45-2342) (BD Pharmingen) for 1 h at 4 °C. 7-AAD (BD Pharmingen) was applied to exclude nonviable cells just before starting the analysis. The stained cells were analyzed with a Gallios flow cytometer (Beckman Coulter Inc., CA). Monocytes/macrophages were identified as CD45 + CD11b + Lineage (B220/CD90.2/CD49b/NK1.1/Ly-6G)- Ly-6Chigh/low cells.

### Quantitative real-time PCR

RNA was extracted from cultured macrophages with an Illustra RNA Spin Mini Kit (GE Healthcare, Chicago, IL), and cDNA was synthesized with a PrimeScript RT Reagent Kit (TAKARA BIO Inc., Shiga, Japan) according to the manufacturer’s instructions. Real-time PCR was performed with TB Green Premix Ex Taq II (TAKARA BIO Inc.) and a StepOnePlus Real-time PCR System (Applied Biosystems, Waltham, MA). The primer sequences are listed in Table S2 in the Data Supplement.

### Evaluation of CX3CR1 and CCR2 expression

Bone marrow cells harvested from CX3CR1-GFP CCR2-RFP mice were cultured for 5 days and then pretreated with 10 µM cholesterol or 7-KC for 24 h before stimulation with 10 ng/mL LPS and 10 ng/mL IFN-γ for 24 h to induce differentiation into inflammatory macrophages. After nuclear counterstaining (Hoechst 33342, Thermo Fisher Scientific Inc.), CX3CR1-positive cells (GFP-positive) and CCR2-positive cells (RFP-positive) were observed under a confocal laser scanning microscope (FV1000, Olympus, Tokyo, Japan).

### Chemotaxis assay

The chemotactic activity of macrophages was measured in a 96-well chemotaxis chamber with a 5-μm pore-sized membrane (ChemoTx, Neuro Probe Inc., Gaithersburg, MD) according to the manufacturer’s instructions. MCP-1 in RPMI-1640 medium was added to the lower chambers, and peritoneal macrophages harvested from WT mice fed the WD or 7KWD for 3 weeks (1 × 10^7^ cells per mL) were placed in the upper chambers. After a 4-h incubation at 37 °C, the upper surface of the membrane was washed with PBS, and 20 mM ethylenediaminetetraacetic acid (EDTA) was applied. After a 20-min incubation at 4 °C, the added EDTA was removed and centrifuged. The migrated cells in the lower chambers were counted.

### RNA sequencing

Peritoneal macrophages were stimulated with 10 µM cholesterol or 7-KC for 24 h, and then RNA was extracted with ISOGEN (Nippon Gene, Tokyo, Japan). RNA-Seq was performed using an Illumina NextSeq 500 (Illumina Inc., Tokyo, Japan). Among all detected genes, differentially expressed transcripts between the cholesterol group and the 7-KC group were chosen according to the stated criteria (fold change > 2 or < 0.5 and FDR < 0.05). The obtained data were analyzed using STRING software (version 11.5) and iDEP software (version 0.94).

### NF-κB activity assay

NF-κB activity was evaluated by measuring p65 translocation into the nucleus using a TransAM NF-κB p65 Kit (Active Motif, Carlsbad, CA) according to the manufacturer’s instructions. Nuclear extracts were extracted from cultured peritoneal macrophages, and protein concentrations were determined with a Bradford-based assay (Pierce Coomassie Plus Assay Kit, Thermo Fisher Scientific Inc.). The nuclear extracts were added to a 96-well plate coated with an oligonucleotide containing the NF-κB consensus sequence (5’-GGGACTTTTCC-3’) and incubated for 1 h at room temperature. An anti-NF-κB antibody was added to each well, and the plate was incubated for 1 h at room temperature. After the plate was incubated for 1 h with a horseradish peroxidase-conjugated secondary antibody, specific binding was detected with a microplate spectrophotometer based on the absorption at 450 nm with a reference wavelength of 655 nm.

### Measurement of mitochondrial ROS

Mitochondrial superoxide was observed and quantified using the mitochondrial superoxide indicator MitoSOX Red (Thermo Fisher Scientific Inc.). After peritoneal macrophages were stimulated with 10 μM 7-KC for 24 h, the cells were incubated with a 2.5 μM MitoSOX solution. After nuclear counterstaining, the fluorescence intensity of MitoSOX was quantified using an FV1000.

### Western blotting

Total protein was extracted from peritoneal macrophages using Cell Lysis Buffer (Cell Signaling Technology Inc., Danvers, MA) according to the manufacturer’s instructions. Protein concentrations were determined with a bicinchoninic acid (BCA) assay (Pierce BCA Protein Assay Kit, Thermo Fish Scientific Inc.). Proteins were separated on 4–20% gradient SDS/polyacrylamide gels and blotted onto polyvinylidene difluoride (PVDF) membranes. The following antibodies were used as primary antibodies: anti-PERK (C33E10, 1:2000), anti-BiP (1:2000), anti-CHOP (D46F1, 1:2000) (Cell Signaling Technology Inc.), and anti-β-actin (AC-15, 1:10,000, Sigma–Aldrich). Anti-Rabbit IgG, HRP-linked whole Ab donkey (GE Healthcare) was used as secondary antibody (1:5000). Chemiluminescence images were acquire with an Image Quant LAS-4010 (GE Healthcare). Densitometric analyses were performed using an Image Quant TL (GE Healthcare).

### Statistical analysis

Data are expressed as the mean ± SEM. Statistical analyses of the differences between two groups were performed using an unpaired t test, and analyses of the differences among three or more groups were performed by one-way or two-way ANOVA, followed by Tukey’s post-hoc multiple comparison test using Prism Software version 8.0 (GraphPad Software, San Diego, CA). Statistical analyses of flow cytometry data were performed using the chi-square test. P values less than 0.05 were considered statistically significant.

## Supplementary Information

Supplementary Information 1.

Supplementary Information 2.

Supplementary Information 3.

## Data Availability

All RNA sequencing data are available at NCBI Sequence Read Archive (SRA) with the project accession number PRJNA810596.
