# Supplementary material for: Dietary 7-ketocholesterol exacerbates myocardial ischemia–reperfusion injury in mice through monocyte/macrophage-mediated inflammation
Source: Sci Rep. 2022 Sep 1;12:14902. doi: 10.1038/s41598-022-19065-z (PMC9436973; doi:10.1038/s41598-022-19065-z)
Supplement: Supplementary file 1 — Supplementary Information 1. [file 41598_2022_19065_MOESM1_ESM.pdf]

Supplementary Figure S1

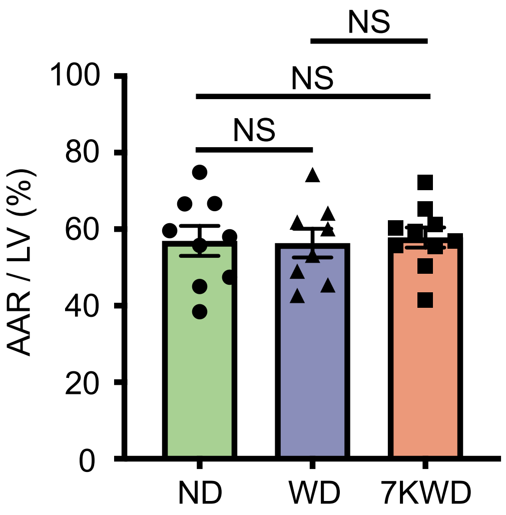

Supplementary Figure S1. Dietary 7-KC did not increase AAR compared to LV after myocardial IR. The AAR / LV quantified 24 hours after IR. N=8-10. The data were analyzed by one-way ANOVA, followed by Tukey's post-hoc multiple comparison test. AAR, area at risk; LV, left ventricle; ND, normal laboratory diet; WD, western diet; 7KWD, WD containing 7-KC.

Supplementary Figure S2

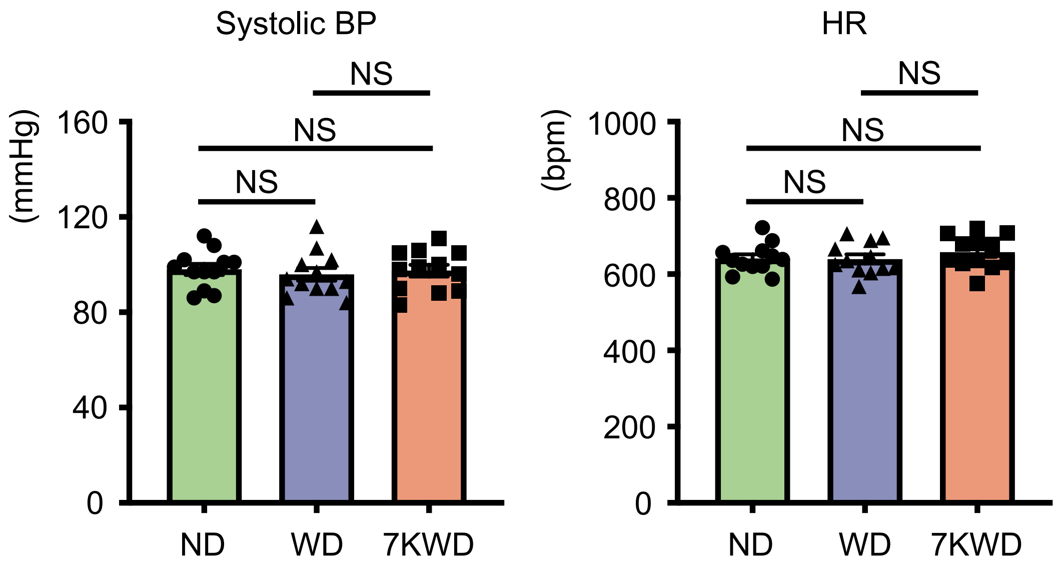

Supplementary Figure S2. Dietary 7-KC did not affect BP or HR. BP and HR measured in mice fed a ND, WD or 7KWD for 3 weeks. N=12. The data were analyzed by one-way ANOVA, followed by Tukey's post-hoc multiple comparison test. BP, blood pressure; HR, heart rate; ND, normal laboratory diet; WD, western diet; 7KWD, WD containing 7-KC.

Supplementary Figure S3

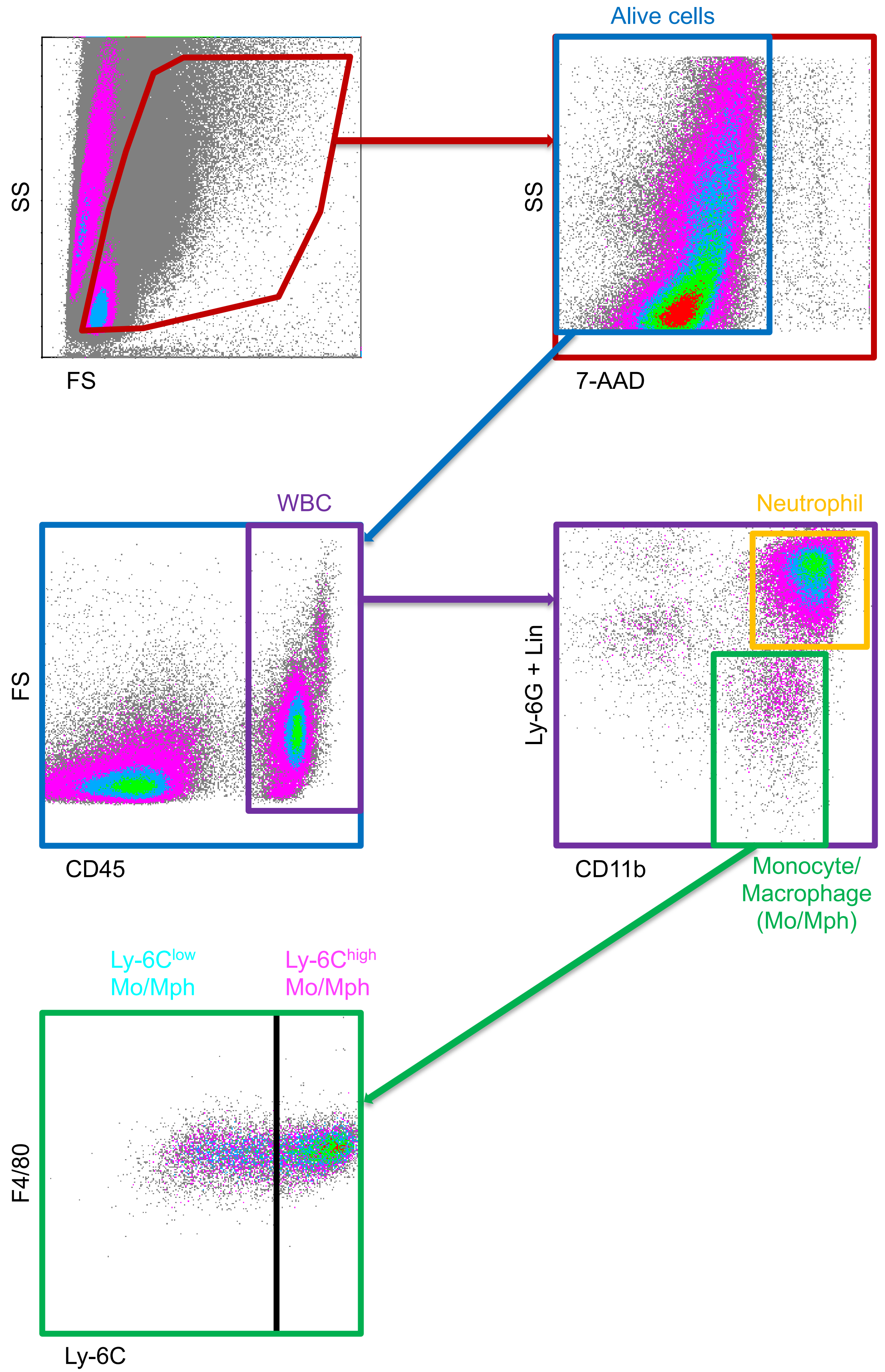

Supplementary Figure S3. Flowcytometric gating strategy. Flowcytometric gating strategy for isolated cells from the heart after myocardial IR. FS, forward scatter; SS, side scatter; WBC, white blood cell; Lin, Lineage; Mo, monocyte; Mph, macrophage.

Supplementary Figure S4

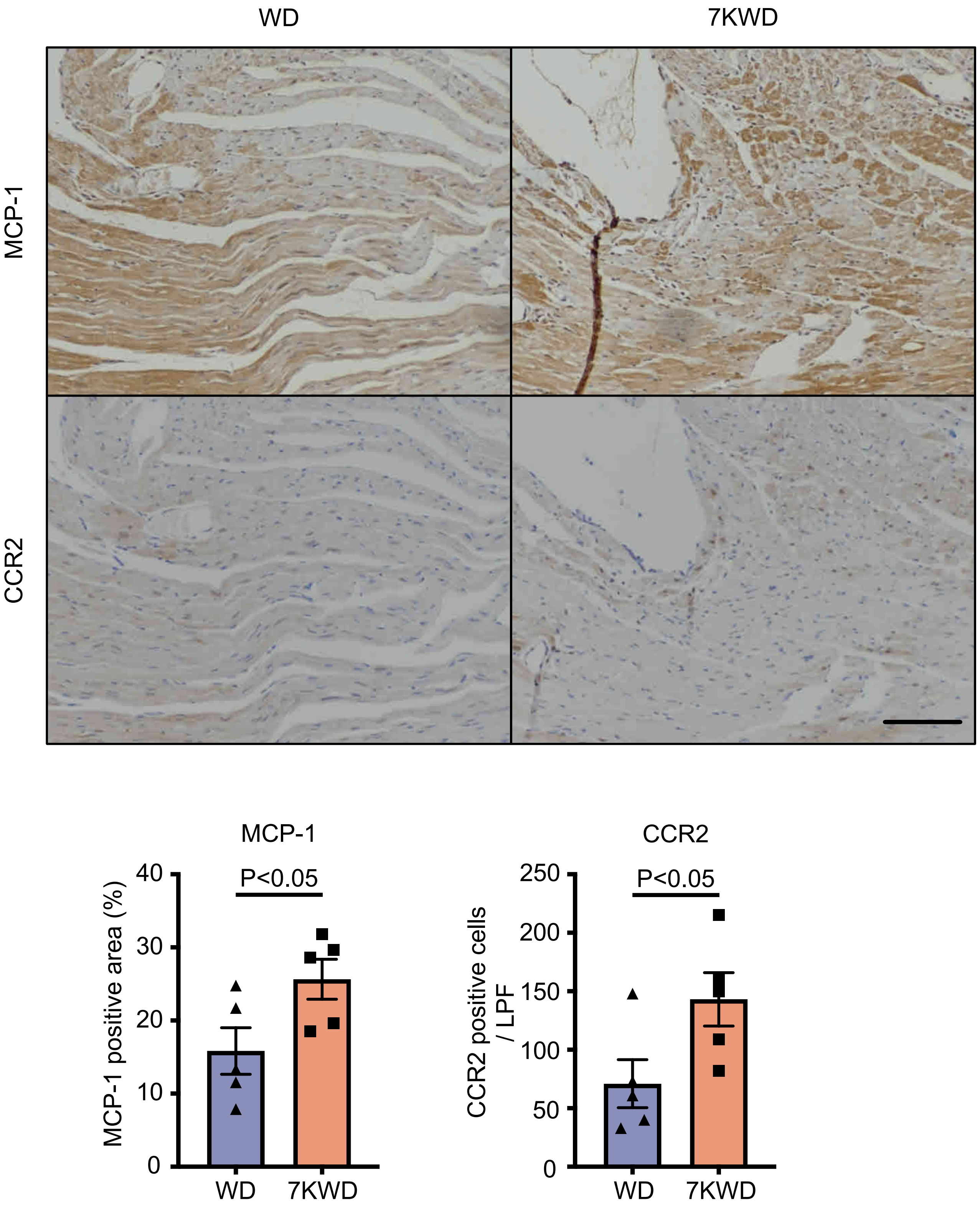

Supplementary Figure S4. Dietary 7-KC increased MCP-1 expression and CCR2-positive cells in the heart after myocardial IR. MCP-1 expression and CCR2-positive monocytes/macrophages in the heart at 24 hours after IR. Scale bar, 100 µm. N=5. The data were analyzed by unpaired t test.

Supplementary Figure S5

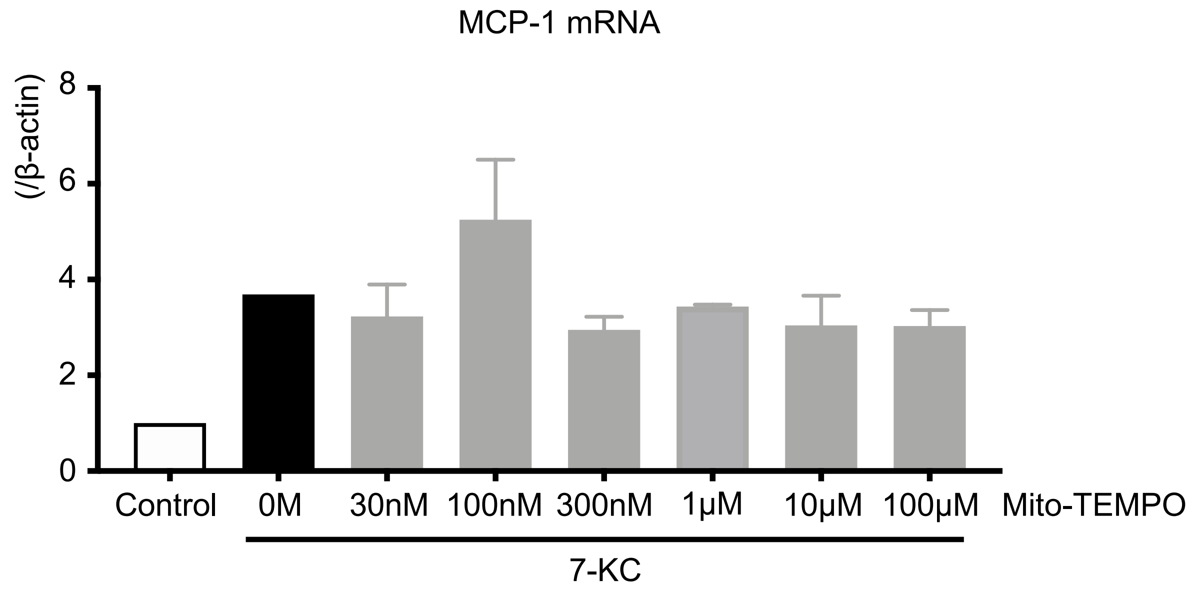

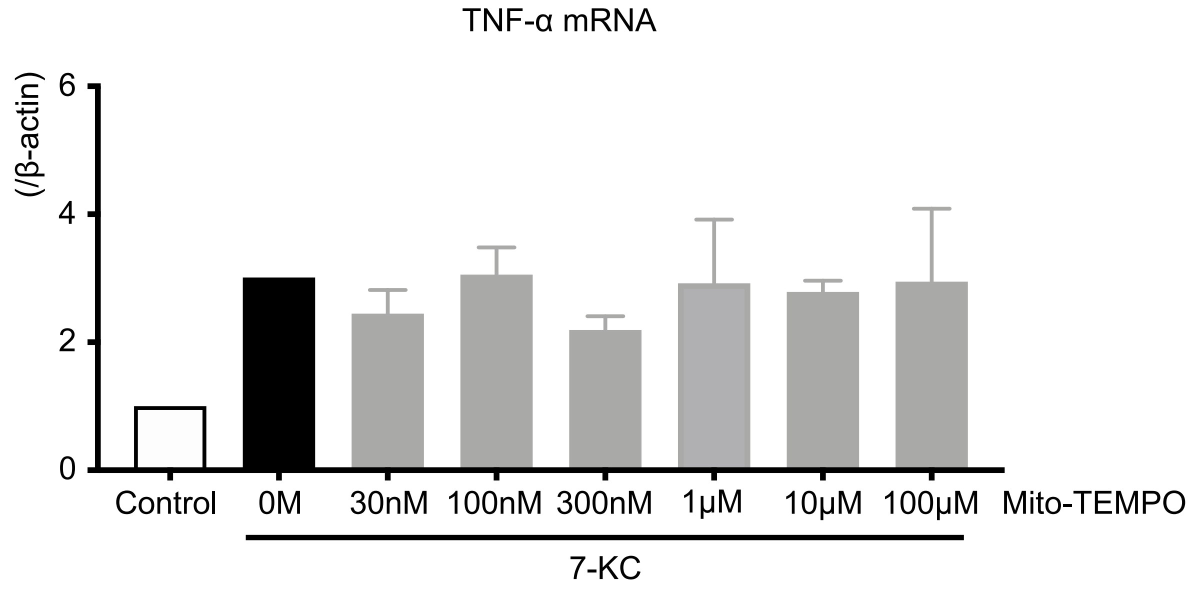

Supplementary Figure S5. Mito-TEMPO did not suppress 7-KC-induced inflammatory gene expression. Peritoneal macrophages were pretreated with 0 to 100 μM Mito-TEMPO for 1 hour before 10 μM 7-KC treatment for 24 hours. Expression of cytokines were quantified by RT-PCR. N=1-2. Control, vehicle control; 7-KC, 7-ketocholesterol

Supplementary Figure S6

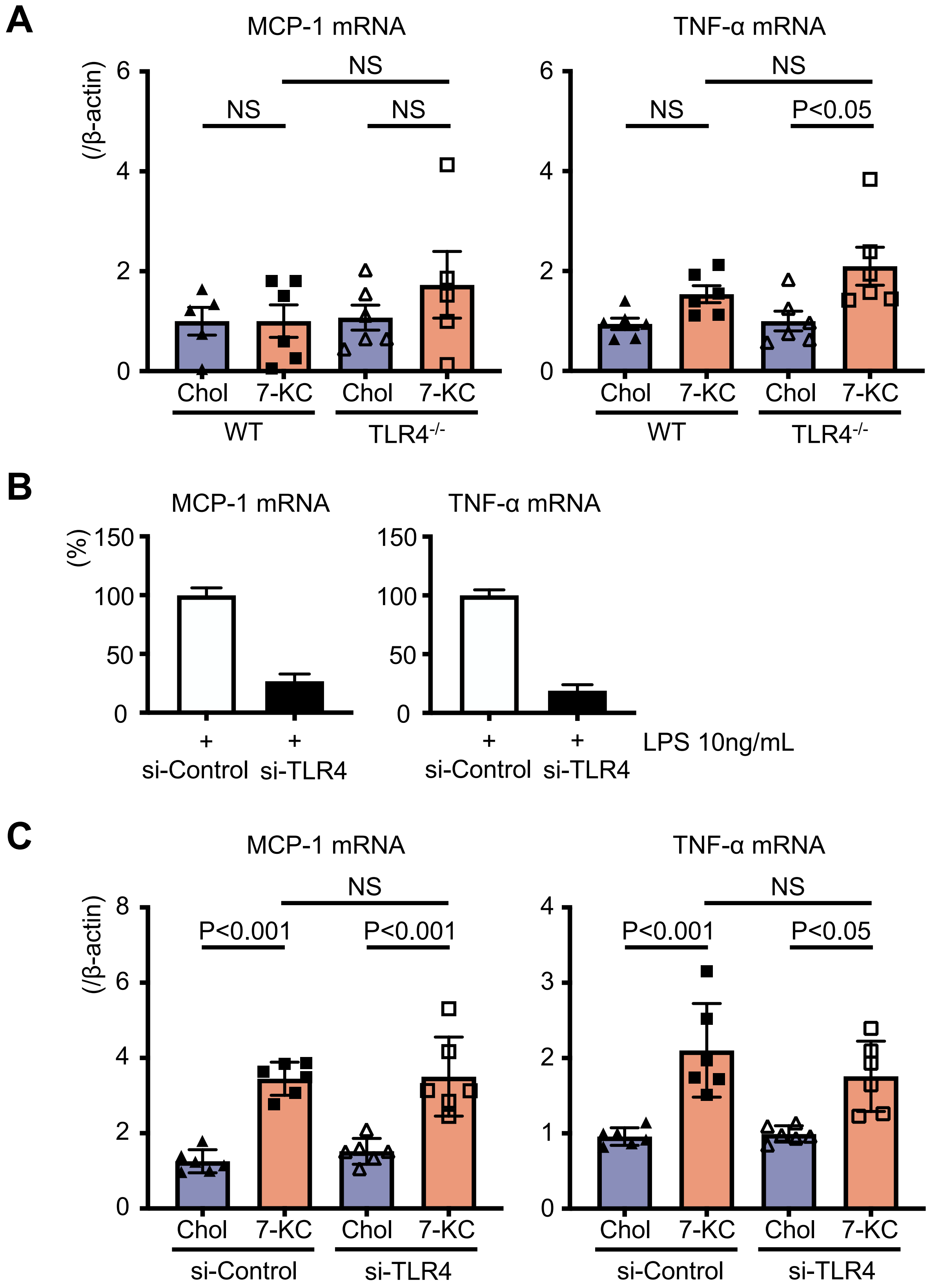

Supplementary Figure S6. 7-KC increased inflammatory cytokines through TLR4-independent pathway. A, mRNA expression of cytokines in peritoneal macrophages isolated from TLR4-deficient mice and treated with 10 μM cholesterol or 7-KC for 24 hours. N=5-6. B, LPS-induced mRNA expression of cytokines in peritoneal macrophages transfected with 5 nM siRNA against TLR4 packaged in LNPs. N=2. C, After transfected with 5 nM siRNA against TLR4, mRNA expression of cytokines in peritoneal macrophages treated with 10 μM cholesterol or 7-KC for 24 hours were quantified by RT–PCR. N=6. The data were analyzed by one-way ANOVA, followed by Tukey's post-hoc multiple comparison test. TLR4, toll-like receptor 4; Chol, cholesterol; 7-KC, 7-ketocholesterol; WT, wild-type mice; TLR4-/-, TLR4-deficient mice; si-Control, nontargeted control siRNA; si-TLR4, siRNA against TLR4; LPS, lipopolysaccharide; LNP, lipid nanoparticle.

Supplementary Figure S7

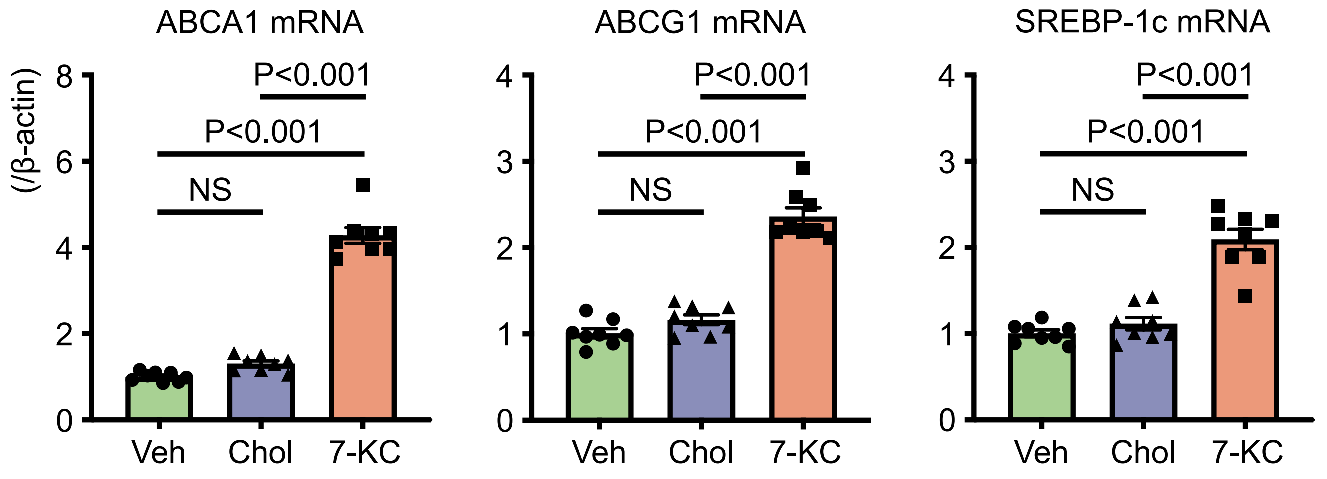

Supplementary Figure S7. 7-KC increased LXR target genes. mRNA expression of LXR target genes related to lipid metabolism in peritoneal macrophages treated with vehicle, 10 μM cholesterol or 7-KC for 24 hours. N=8. The data were analyzed by one-way ANOVA, followed by Tukey's post-hoc multiple comparison test. Veh, vehicle; Chol, cholesterol; 7-KC, 7-ketocholesterol; ABCA1, ATP-binding cassette protein A1; ABCG1, ATP-binding cassette protein G1; SREBP-1c, sterol regulatory element-binding protein 1c; LXR, liver X receptor.

Supplementary Figure S8

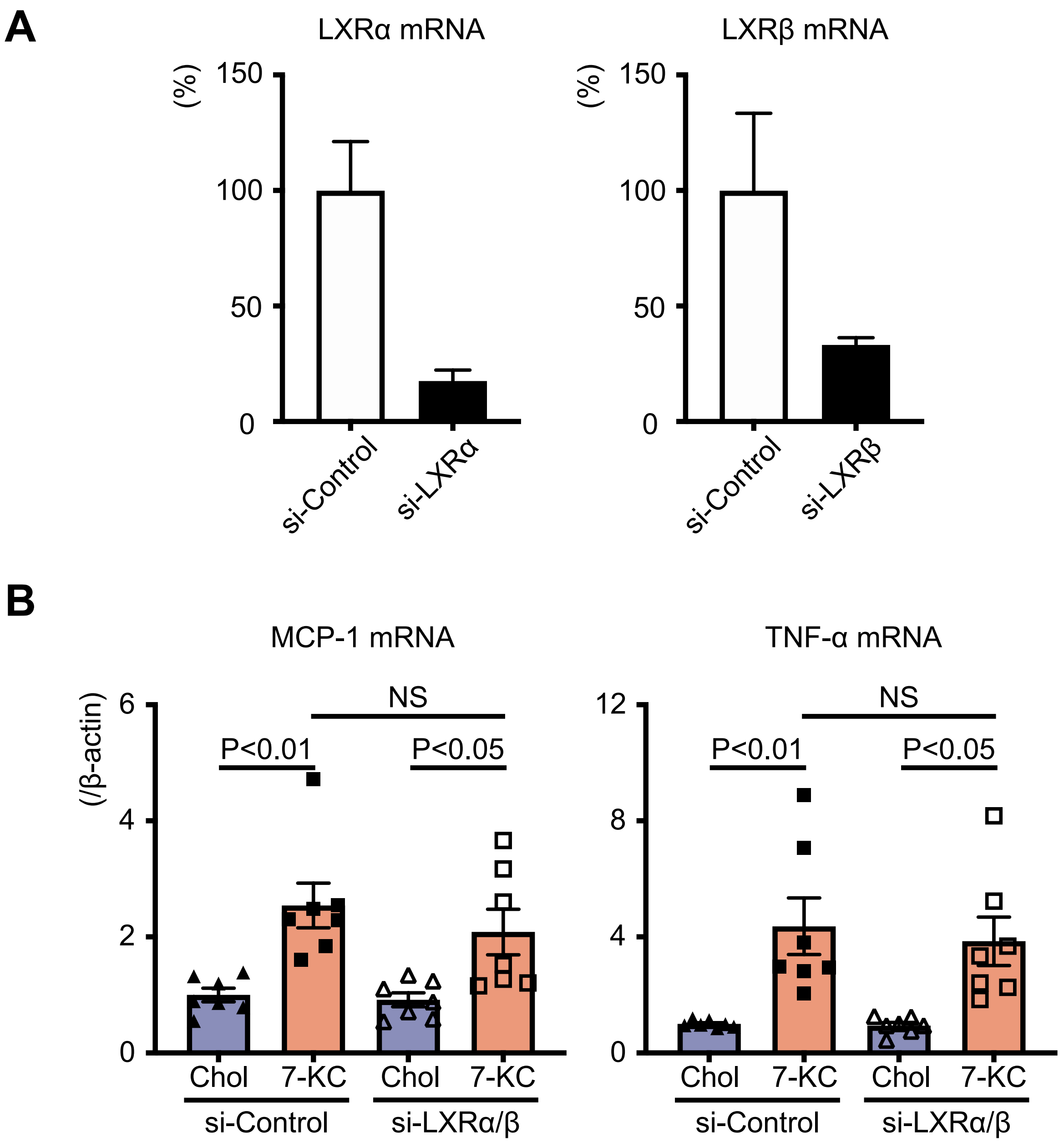

Supplementary Figure S8. Silencing LXR did not inhibit 7-KC-induced proinflammatory cytokines. A, Peritoneal macrophages were transfected with 10 nM siRNA against LXRα or LXRβ packaged in LNPs for 24 hours. Twelve hours after transfection was completed, expression of target genes were quantified by RT–PCR. N=2. B, After transfected with 10 nM siRNA against LXRα and LXRβ, mRNA expression of cytokines in peritoneal macrophages treated with 10 μM cholesterol or 7-KC for 24 hours were quantified by RT–PCR. N=7. The data were analyzed by one-way ANOVA, followed by Tukey's post-hoc multiple comparison test. LXR, liver X receptor; si-Control, nontargeted control siRNA; si-LXRα, siRNA against LXRα; si-LXRβ, siRNA against LXRβ; si-LXRα/β, siRNA against LXRα and LXR β; LNP, lipid nanoparticle; Chol, cholesterol; 7-KC, 7-ketocholesterol.

Supplementary Figure S9

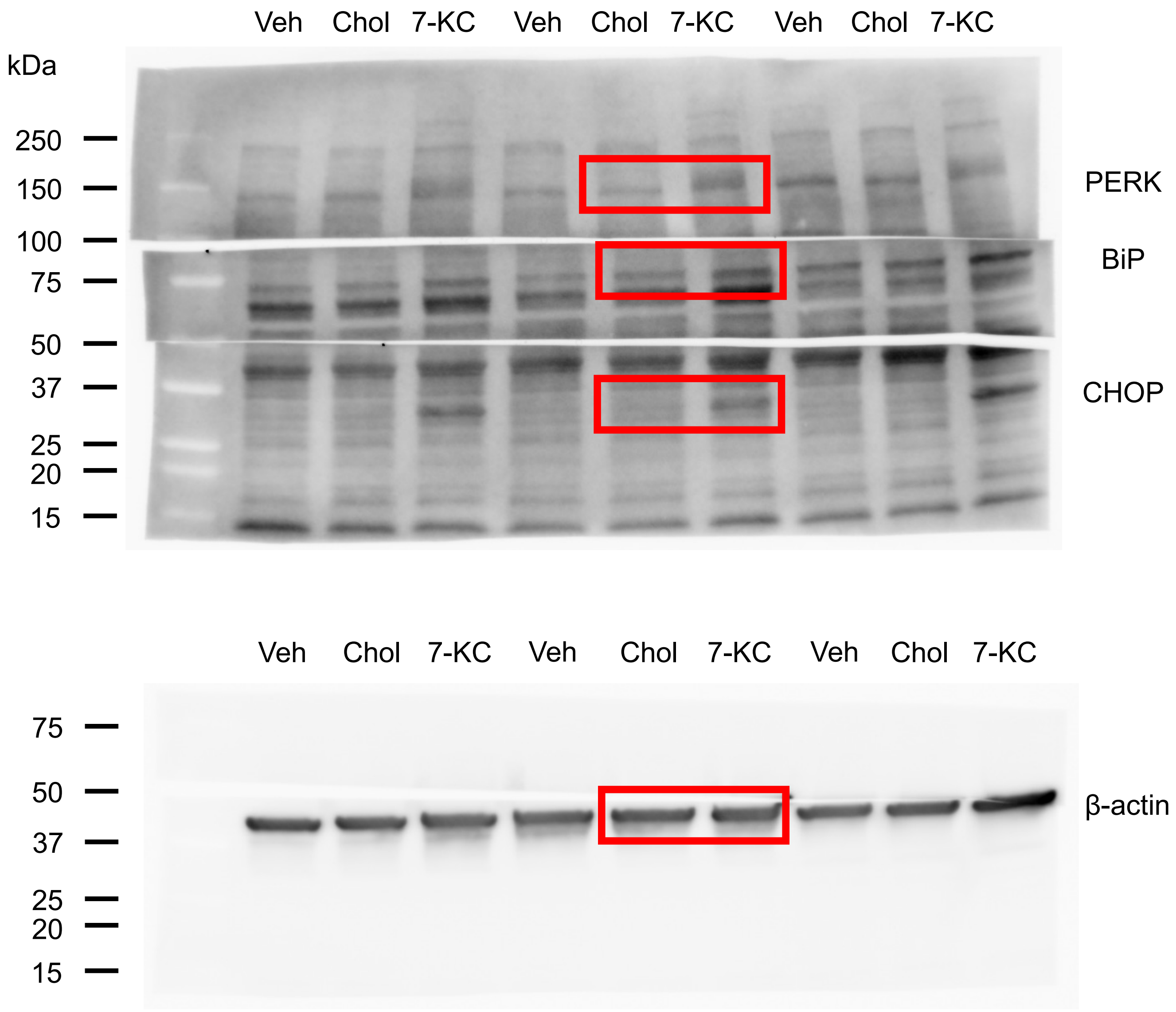

Supplementary Figure S9. Original blots/gels of Figure 5A. We cannot provide images of full-length blots because we cut membranes before hybridization with antibodies. Veh, vehicle; Chol, cholesterol; 7-KC, 7-ketocholesterol; PERK, PKR-like endoplasmic reticulum kinase; BiP, binding immunoglobulin protein; CHOP, C/EBP homologous protein.

Supplementary Figure S10

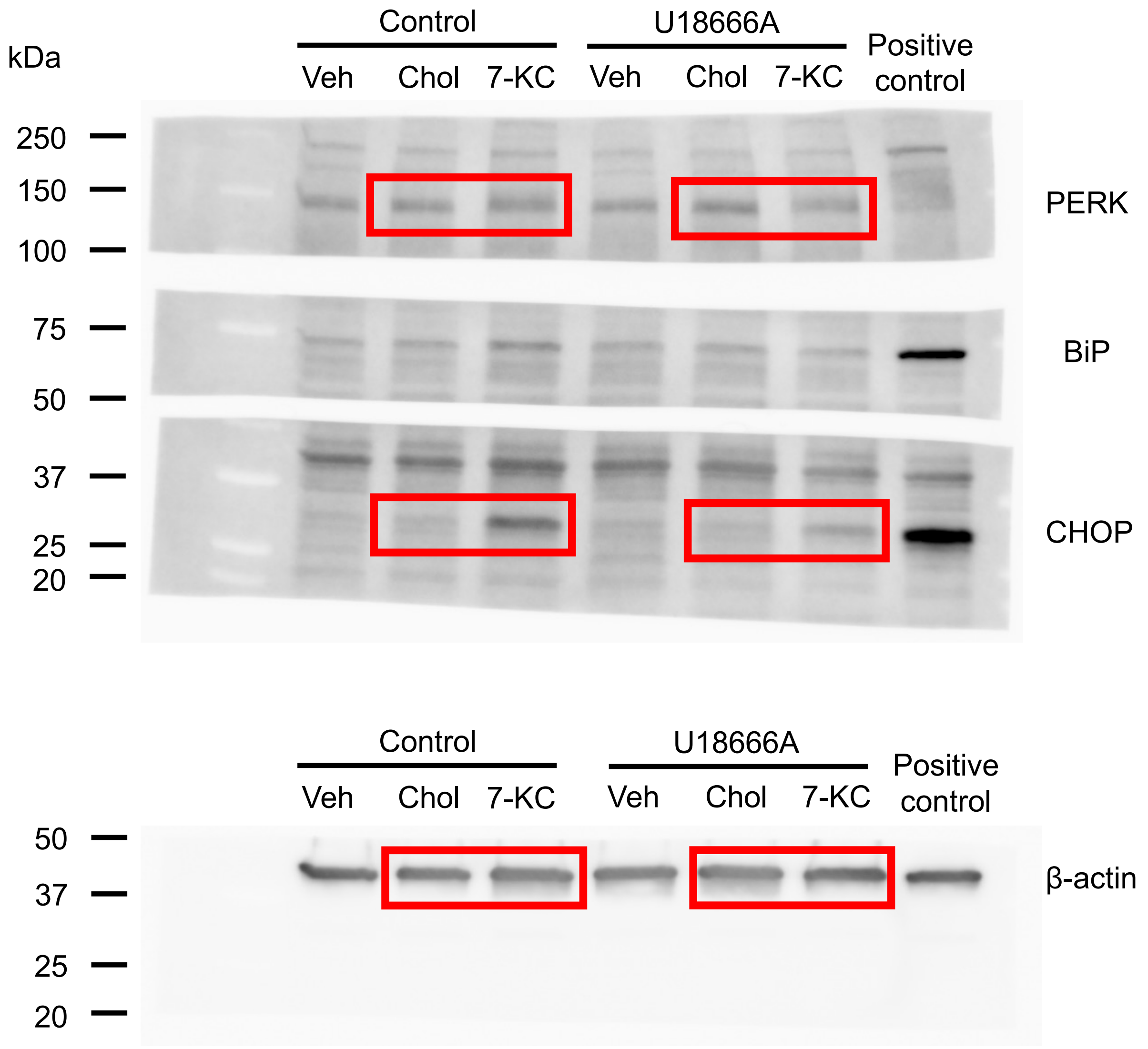

Supplementary Figure S10. Original blots/gels of Figure 5B. We cannot provide images of full-length blots because we cut membranes before hybridization with antibodies. Veh, vehicle; Chol, cholesterol; 7-KC, 7-ketocholesterol; PERK, PKR-like endoplasmic reticulum kinase; BiP, binding immunoglobulin protein; CHOP, C/EBP homologous protein.

Supplementary Figure S11

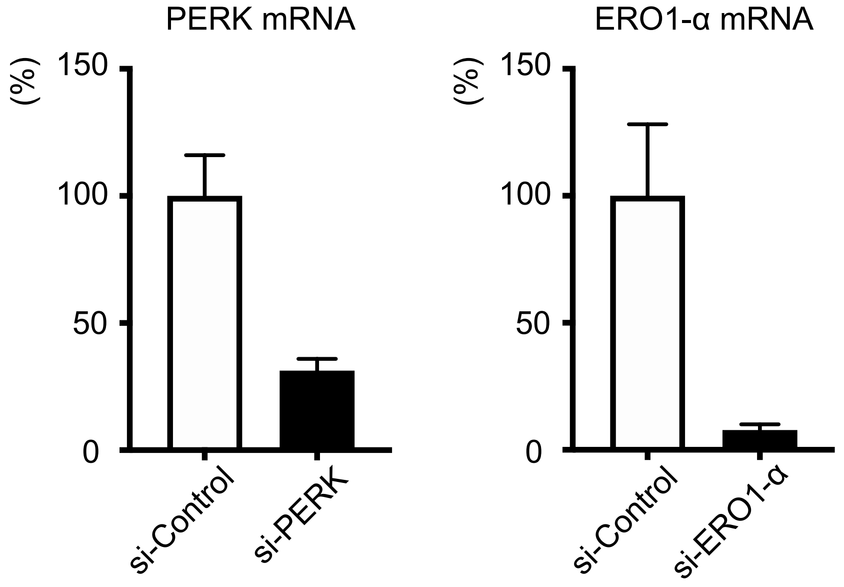

Supplementary Figure S11. PERK and ERO1-α siRNA suppressed each target gene. Peritoneal macrophages were transfected with 25 nM siRNA against PERK or ERO1-α packaged in LNPs for 24 hours. Twelve hours after transfection was completed, expression of target genes were quantified by RT–PCR. N=3. PERK, PKR-like endoplasmic reticulum kinase; ERO1-α, endoplasmic reticulum oxidase 1 α; si-Control, nontargeted control siRNA; si-PERK, siRNA against PERK; si-ERO1-α, siRNA against ERO1-α; LNP, lipid nanoparticle.

Supplementary Table S1. Pathway analyses of RNA-Seq

KEGG Pathways			
**#Term ID**	**Term description**	**Count in gene set**	**FDR**
**mmu00100**	Steroid biosynthesis	9 of 19	2.96E-15
**mmu01100**	Metabolic pathways	17 of 1296	2.03E-06
**mmu04657**	IL-17 signaling pathway	6 of 91	9.34E-06
**mmu05134**	Legionellosis	5 of 57	2.02E-05
**mmu04141**	Protein processing in endoplasmic reticulum	6 of 161	0.00014
**mmu00900**	Terpenoid backbone biosynthesis	3 of 22	0.00082
**mmu04621**	NOD-like receptor signaling pathway	5 of 164	0.0016
**mmu04668**	TNF signaling pathway	4 of 108	0.0035
**mmu04060**	Cytokine-cytokine receptor interaction	5 of 252	0.0073
**mmu05418**	Fluid shear stress and atherosclerosis	4 of 140	0.0073
**mmu01230**	Biosynthesis of amino acids	3 of 75	0.0122
**mmu04612**	Antigen processing and presentation	3 of 78	0.0126
**mmu04064**	NF-kappa B signaling pathway	3 of 93	0.019
**mmu00650**	Butanoate metabolism	2 of 27	0.0223
**mmu04931**	Insulin resistance	3 of 108	0.0245
**mmu05145**	Toxoplasmosis	3 of 107	0.0245
**mmu00250**	Alanine, aspartate and glutamate metabolism	2 of 36	0.0284
**mmu01200**	Carbon metabolism	3 of 118	0.0284
**mmu04380**	Osteoclast differentiation	3 of 122	0.0284
**mmu05020**	Prion diseases	2 of 34	0.0284
**mmu04210**	Apoptosis	3 of 135	0.0335
**mmu05144**	Malaria	2 of 45	0.0375
**mmu04010**	MAPK signaling pathway	4 of 292	0.0412
**mmu01212**	Fatty acid metabolism	2 of 51	0.0435
**mmu00280**	Valine, leucine and isoleucine degradation	2 of 55	0.0481
**mmu05164**	Influenza A	3 of 165	0.0481

KEGG pathways were identified using the KEGG pathway database (www.kegg.jp/kegg/kegg1.html). RNA-Seq, RNA sequencing; KEGG, Kyoto Encyclopedia of Genes and Genomes; FDR, false discovery rate.

Supplementary Table S2. qPCR primer information

Gene	Forward	Reverse
Mus musculus		
Actb	CCTGAGCGCAAGTACTCTGTGT	GCTGATCCACATCTGCTGGAA
Il1b	TCCAGGATGAGGACATGAGCAC	GAACGTCACACACCAGCAGGTTA
Il6	TCCACGATTTCCCAGAGAAC	AGTTGCCTTCTTGGGACTGA
Ccl2	AGGTCCCTGTCATGCTTCTG	TCTGGACCCATTCCTTCTTG
Tnf	AGCCCCCAGTCTGTATCCTT	CTCCCTTTGCAGAACTCAGG
Nos2	CCCCGCTACTACTCCATCAG	CCACTGACACTTCGCACAAA
Abca1	AACAGTTTGTGGCCCTTTTG	AGTTCCAGGCTGGGGTACTT
Abcg1	GTACCATGACATCGCTGGTG	AGCCGTAGATGGACAGGATG
Srebf1	GATCAAAGAGGAGCCAGTGC	TAGATGGTGGCTGCTGAGTG
Nr1h3	CATCTTCTCTGCAGACCGGC	TAGCATCCGTGGGAACATCA
Nr1h2	CCCAAAGTCACGCCCTGG	CCACAATCTCCTGGACCGAG
Eif2ak3	GGTCTGGTTCCTTGGTTTCA	TTCGCTGGCTGTGTAACTTG
Ero1l	GCCAGGTTAGTGGTTACTTGG	GGCCTCTTCAGGTTTACCTTGT
