# Supplementary material for: Dietary 7-ketocholesterol exacerbates myocardial ischemia–reperfusion injury in mice through monocyte/macrophage-mediated inflammation
Source: Sci Rep. 2022 Sep 1;12:14902. doi: 10.1038/s41598-022-19065-z (PMC9436973; doi:10.1038/s41598-022-19065-z)
Supplement: Supplementary file 2 — Supplementary Information 2. [file 41598_2022_19065_MOESM2_ESM.pdf]

Supplementary Figure S9
Membrane 1

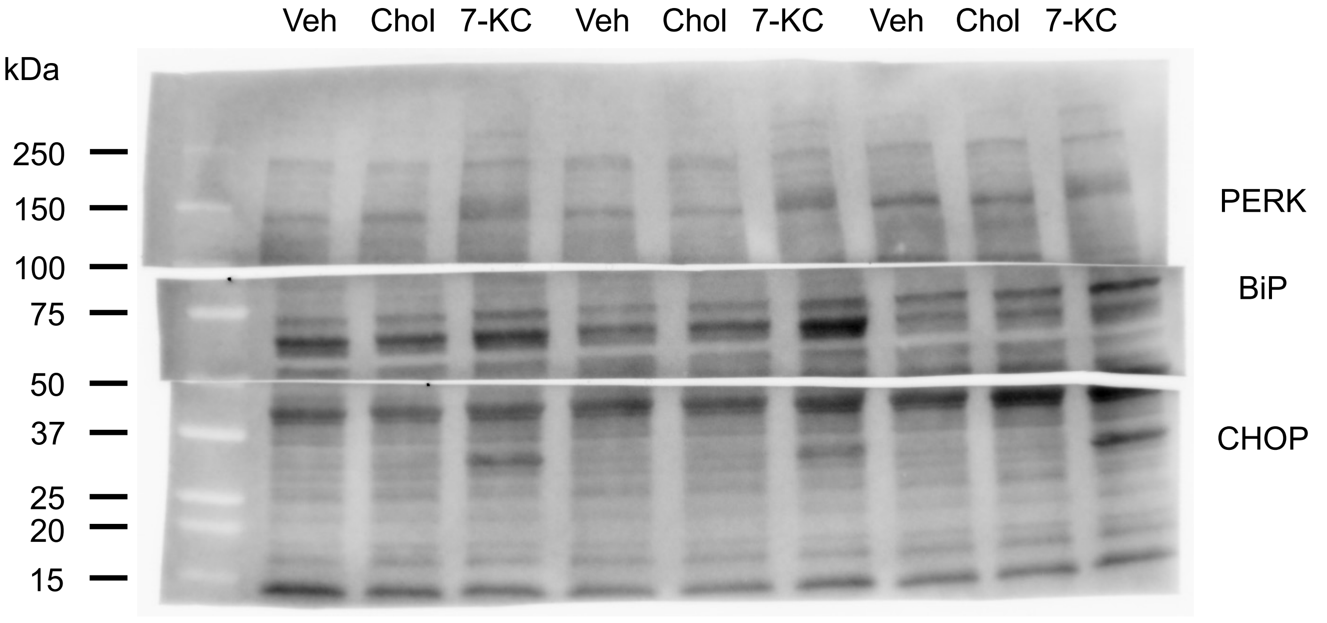

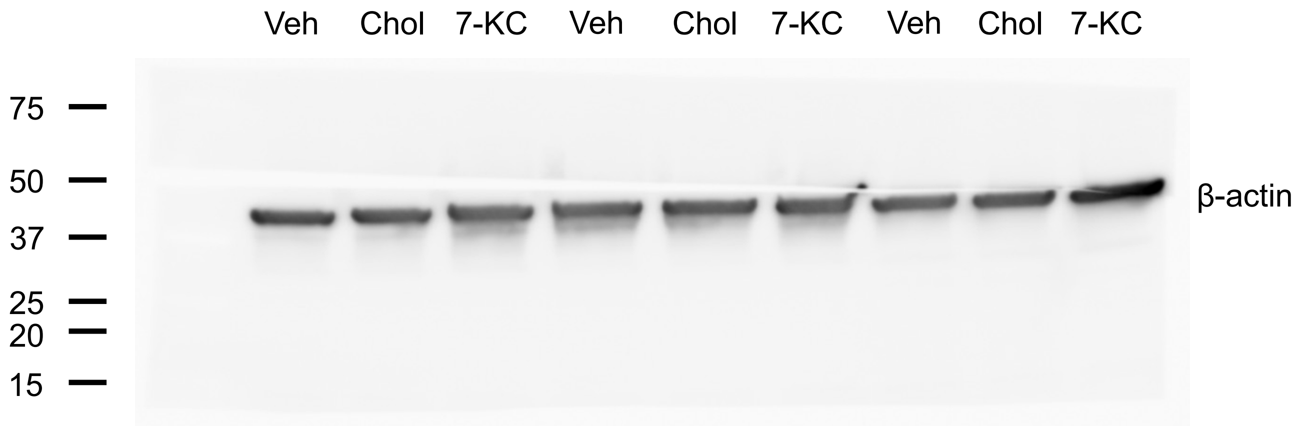

Veh, vehicle; Chol, cholesterol; 7-KC, 7-ketocholesterol; PERK, PKR-like endoplasmic reticulum kinase; BiP, binding immunoglobulin protein; CHOP, C/EBP homologous protein.

Supplementary Figure S9
Membrane 2

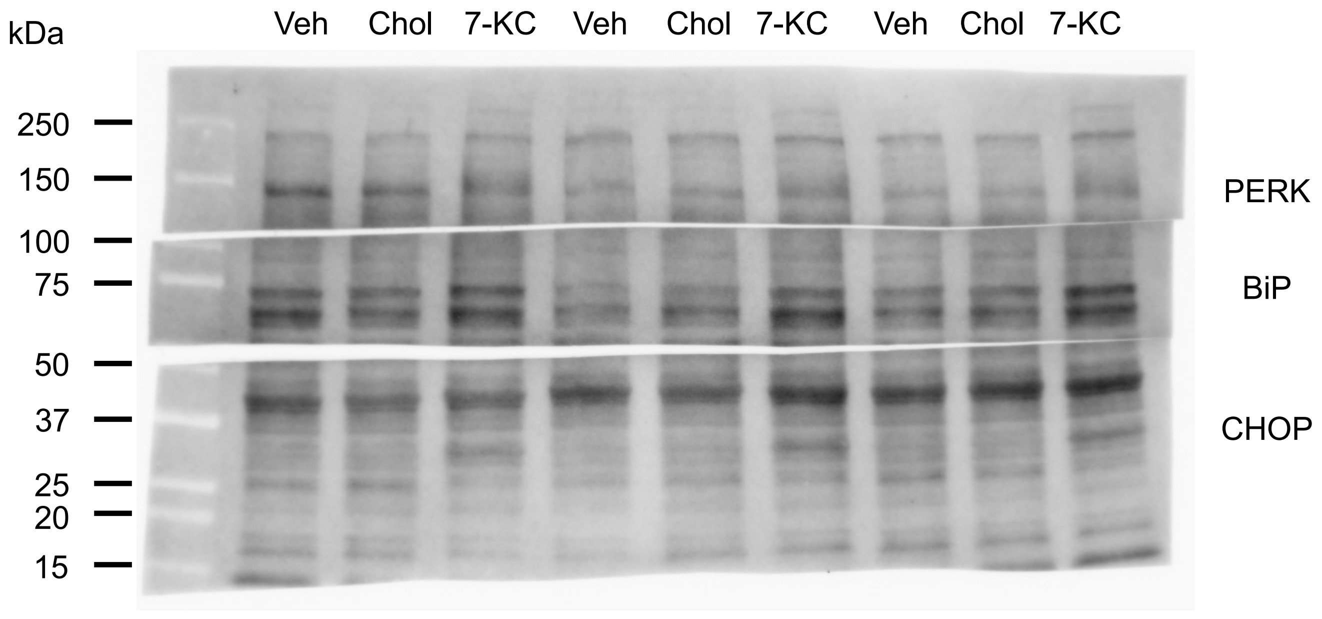

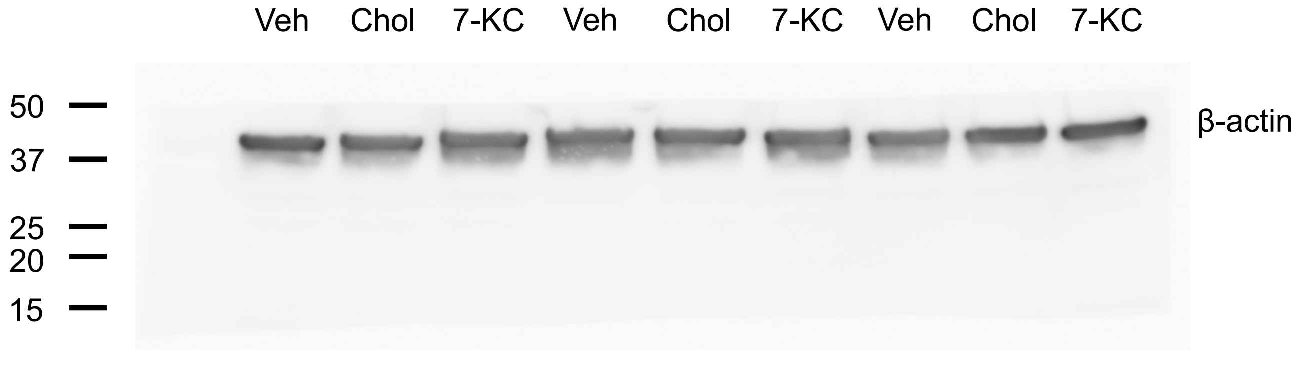

Veh, vehicle; Chol, cholesterol; 7-KC, 7-ketocholesterol; PERK, PKR-like endoplasmic reticulum kinase; BiP, binding immunoglobulin protein; CHOP, C/EBP homologous protein.

Supplementary Figure S9
Membrane 3

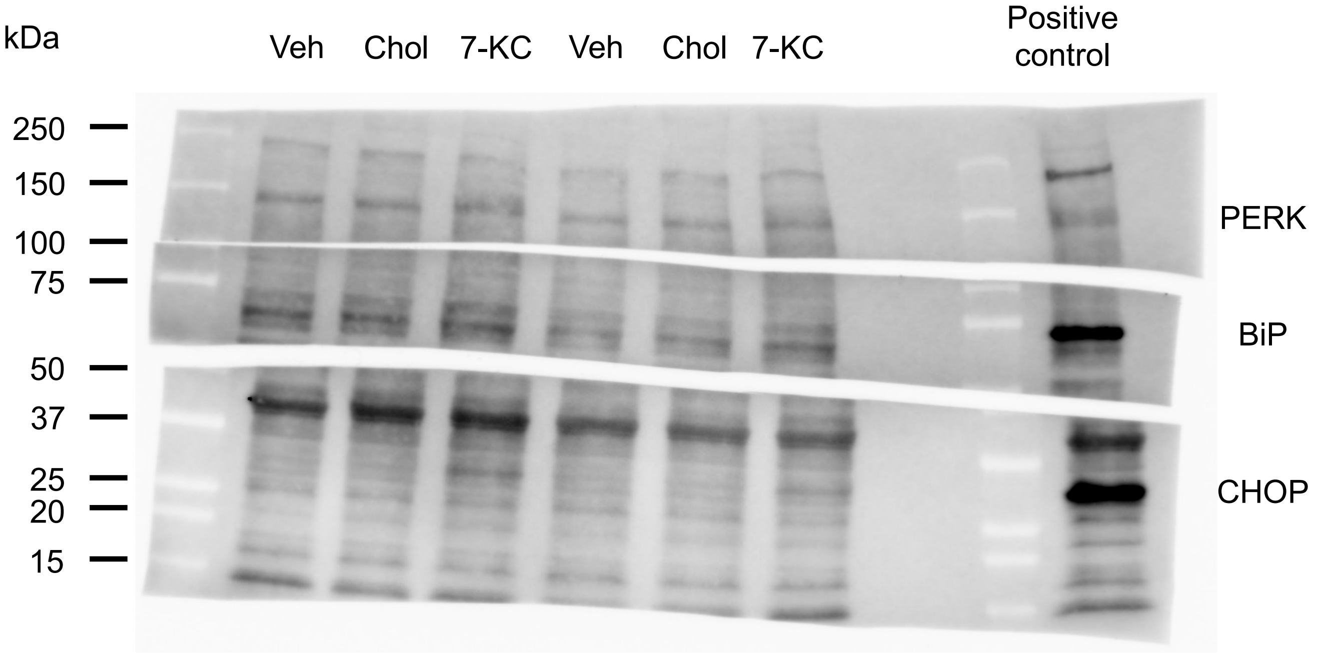

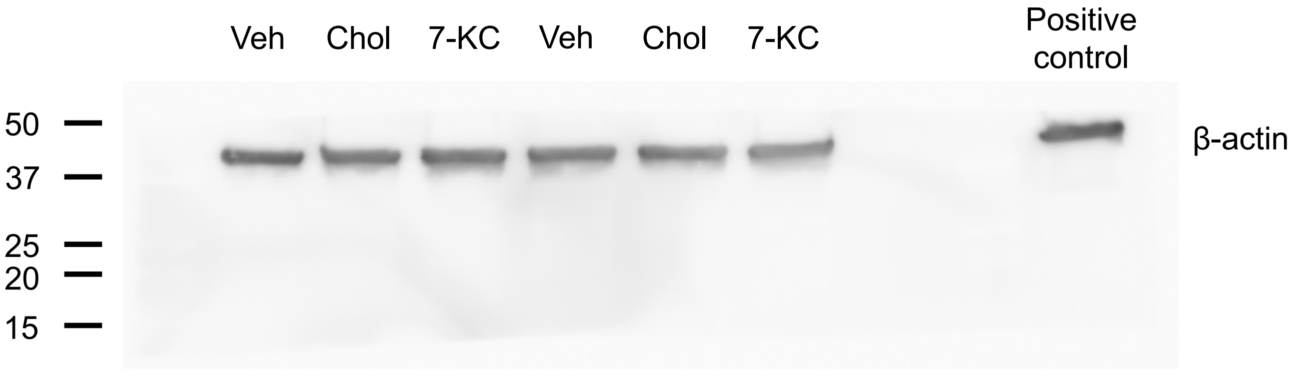

Veh, vehicle; Chol, cholesterol; 7-KC, 7-ketocholesterol; PERK, PKR-like endoplasmic reticulum kinase; BiP, binding immunoglobulin protein; CHOP, C/EBP homologous protein.
